# Tracing the birth of structural domains from loops during protein evolution

**DOI:** 10.1038/s41598-023-41556-w

**Published:** 2023-09-06

**Authors:** M. Fayez Aziz, Fizza Mughal, Gustavo Caetano-Anollés

**Affiliations:** 1 Evolutionary Bioinformatics Laboratory, Department of Crop Sciences, University of Illinois, Urbana, IL 61801 USA; 2 C.R. Woese Institute for Genomic Biology, University of Illinois, Urbana, IL 61801 USA

**Keywords:** Molecular evolution, Computational biology and bioinformatics, Network topology

## Abstract

The structures and functions of proteins are embedded into the loop scaffolds of structural domains. Their origin and evolution remain mysterious. Here, we use a novel graph-theoretical approach to describe how modular and non-modular loop prototypes combine to form folded structures in protein domain evolution. Phylogenomic data-driven chronologies reoriented a bipartite network of loops and domains (and its projections) into ‘waterfalls’ depicting an evolving ‘elementary functionome’ (EF). Two primordial waves of functional innovation involving founder ‘*p*-loop’ and ‘winged-helix’ domains were accompanied by an ongoing emergence and reuse of structural and functional novelty. Metabolic pathways expanded before translation functionalities. A dual hourglass recruitment pattern transferred scale-free properties from loop to domain components of the EF network in generative cycles of hierarchical modularity. Modeling the evolutionary emergence of the oldest P-loop and winged-helix domains with AlphFold2 uncovered rapid convergence towards folded structure, suggesting that a folding vocabulary exists in loops for protein fold repurposing and design.

## Introduction

“… I arrive now at the ineffable core of my story. And here begins my despair as a writer. All language is a set of symbols whose use among its speakers assumes a shared past. How, then, can I translate into words the limitless Aleph, which my floundering mind can scarcely encompass? …”— Jorge Luis Borges, The Aleph and Other Stories

The protein world is both structured and functionally complex. Its emergence and history merits exploration. The evolutionary principle of spatiotemporal continuity, the ‘*lex continui’* promoted by Leibnitz, requires that structural domains – the structural, functional and evolutionary units of proteins – emerge from earlier structural states. These prior states likely involve a combinatorial origami of dipeptides capable of forming flexible protein loop structures, which led to coevolutionary interactions with nucleic acid cofactors and the rise of genetics^1^. Prior states may also involve evolutionary stable and functionally relevant loop intermediates capable of giving rise to the enormous diversity of protein domains that exist in nature^2,3^. We here explore such scenario of emergence with structural phylogenomics and evolving networks.

Loops define a diverse group of *supersecondary* building blocks made of helix, strand, turn and coil segments that are generally ~ 25 to 30 amino acid residues long, much smaller than the ~ 100 amino acid residues typical of an average compact domain^4,5^. Loop structures embody non-regular (aperiodic) loop regions spanning ‘helical’ and ‘sheet’ structural components^6^, which direct the polypeptide chain in space and are often functionally important. Supersecondary ‘closed loop’ structures collapse into extended flexible or rigid loop-shaped primordial intermediate conformations stabilized by van der Waals locks^7,8^. Loop prototypes are ubiquitous structures regarded as modern determinants of molecular function^9,10^. While their biophysical properties may constrain their evolution, studies identified evolutionarily conserved loop prototypes that were likely responsible for the early rise of molecular functions in protein evolution. A first group of ‘elementary functional loop’ (EFL) prototypes combine with others to form active sites that bind cofactors and exert molecular functions^10–13^. These EFLs were obtained by iterative derivation of sequence profiles from protein coding sequences in complete proteomes using a scoring function that weights profile positions according to their information content^10^. Distant evolutionary relationships between protein functions of EFLs revealed patterns of motif reuse in archaeal proteins^11^. A chronology of bipartite networks linked domains to EFLs (and their projections) and showed that the multifunctional α-β-α layered design typical of P-loop and Rossmann-like sandwich structures was primordial^14^. The networks also showed EFL recruitment events occurring throughout the 3.8 billion years (Gy) history of proteins, suggesting the origin of novel domains is an ongoing process. In contrast with EFLs, a second group of highly repeated non-combinable loop structures present in popular folds indicate remnants of an ancient peptide ‘vocabulary’ that formed folded polypeptides during a primordial RNA-peptide world^15^. Using machine learning methods, biphasic patterns in probability distributions highlighted high-scoring subdomain-sized fragments that were unified by < 30% sequence similarities. These fragments were aligned into folded loop structures that were 9–39 residue long, embedding helix-turn-helix, helix-hairpin-helix, ribosomal protein, P-loop/dinucleotide-binding β-α-β (catalytic), and metal ion/iron-sulfur cluster (binding function) motifs. A third general type of supersecondary structural motifs involve widely reused, contiguous, and non-overlapping segments with longer lengths varying from 35 to 200 amino acids^16,17^. These so-called ‘themes’ have been used to build networks of domains and motifs linked by motif reuse in domains^16^, which interestingly increased with decreasing theme length following a power law^17^. The power law indicates a significantly biased distribution of themes in proteins. All three approaches characterize (1) supersecondary motifs by sequence and/or structure similarities, not necessarily carrying any evolutionary relationship; (2) motif recurrence across proteins driven by biological function; and (3) complex patterns showcasing an interplay of divergent vs. convergent evolution driven by rearrangements, duplications, and divergences. EFLs, loops and themes therefore represent ancient building blocks that are evolutionarily conserved.

We previously reconstructed evolutionary timelines of molecular accretion built with phylogenomic methods from the sequence and structure of thousands of nucleic acid molecules and millions of protein sequences encoded in thousands of genomes [reviewed in^18,19^]. These chronologies showed a gradual evolutionary appearance of domain structures^20,21^, an evolving combinatorial rearrangement of domains in proteins^22^, and gradual accumulation of chemical, biophysical and molecular functions^23,24^. For instance, tracing chemical reaction mechanisms operating in metabolic enzymes uncovered a natural history of biocatalysis^25^. Similarly, tracing the average relative distance of amino acid contacts in the tertiary structure of proteins, a property known as ‘contact order’ that correlates to flexibility, showed that folding speed follows a biphasic pattern of increase and decrease during protein evolution^26^.

We focus on loops sourced from the ArchDB database^27^. ArchDB provides an exhaustive classification of loop structures into loop prototypes (supersecondary motifs) based on both a Density Search (DS) clustering algorithm and a graph-based Markov clustering (MCL) algorithm, both of which are structural alignment (RMSD)-independent. Both implementations explore a multidimensional feature space defined by the number of amino acid residues (length) of aperiodic structure, bracing secondary structures, and the conformation and geometry of loop structures. Our analysis makes use of the more stringent DS ‘mode-seeking’ classification method, which detects regions of feature space with high loop density organized around centroids. The method limits the ‘length’ of loops and enlarges the coverage of clustered groups. We use a graph theoretical approach to trace the coevolutionary history of loop prototypes (simply termed ‘loops’) and protein structural domains defined at the fold family (FF) level of the SCOP domain classification^28^ (termed ‘domains’). Our evolving networks reveal remarkable patterns of emergence at molecular level. They describe how loops of ancient and more recent origin combine to form domain structures in protein evolution. The method allows to model the emergence of the folded structure of domains using ab initio structural prediction.

## Results and discussion

### Reconstructing the history of an ‘elementary functionome’ of loop structures

**Figure 1:**
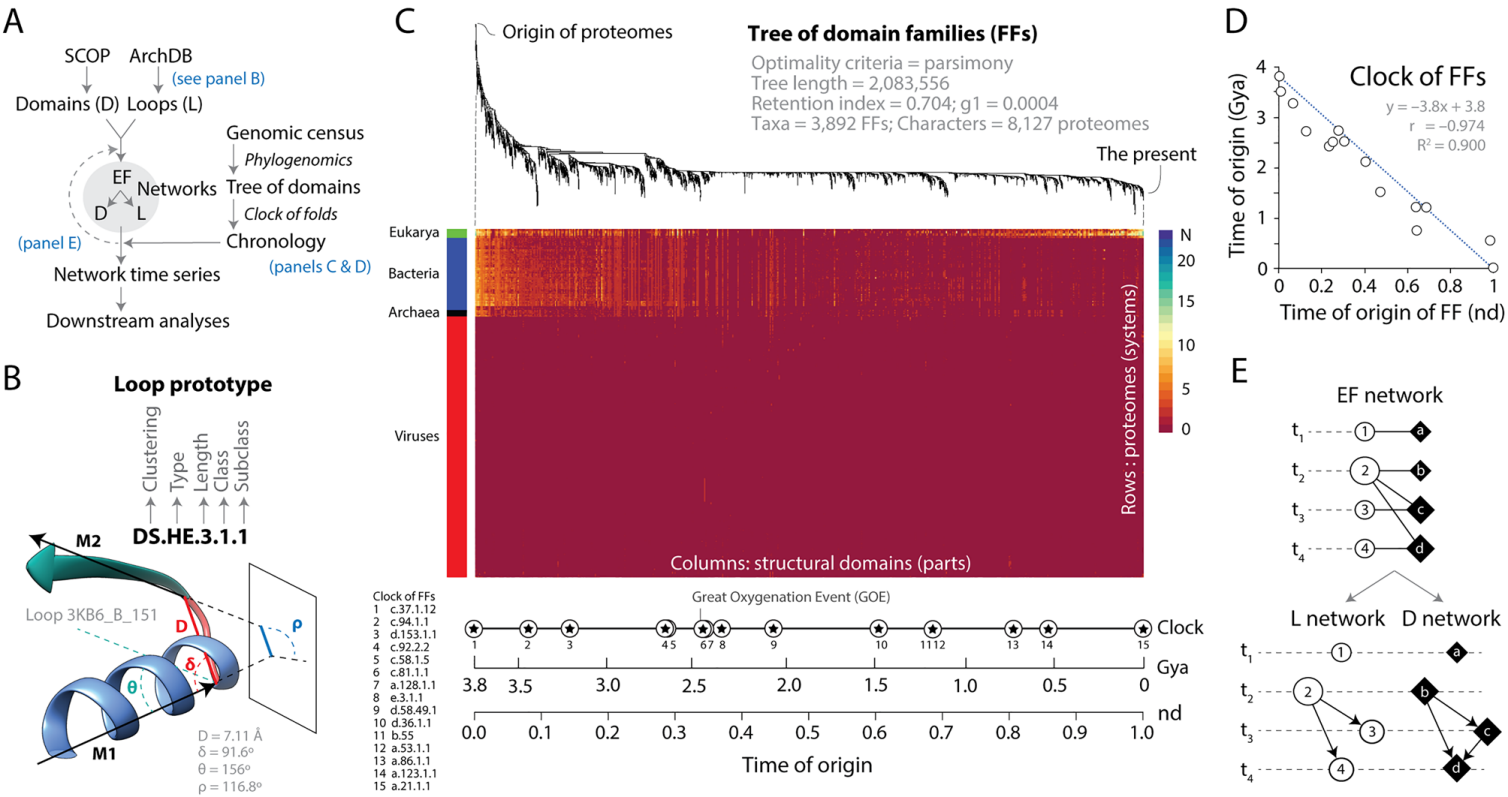
General experimental strategy. (**A**) Workflow describing the generation of time series of a bipartite ‘elementary functionome’ (EF) network and its loop (L) and domain (D) projections. The SCOP^28^ and ArchDB^27^ classifications are used to map loop prototypes to domain families along a chronology built with phylogenomic methodologies. The chronology adds time to network makeup and downstream analysis evaluates network structure (e.g., hierarchy, community structure) and fold emergence with AlphaFold predictions. (**B**) Definition of a loop prototype in ArchDB. The loop is defined by the bracing secondary structures of the loop, the number of residues forming the aperiodic structure, its conformation (ϕ and ψ backbone dihedral angles of the participating residues), and the geometry of the loop. The atomic model of the 3KB6_B_151 loop that is part of prototype DS.HE.3.1.1 shows its geometric properties defined by four internal coordinates (D, δ, θ, ρ) extracted from the orientation of principal vectors (M1 and M2) of bracing secondary structures: D (Distance), the Euclidean distance between the boundaries of the aperiodic structure; Delta (hoist) angle (δ), the angle between M1 and D; Theta (packing) angle (θ), the angle between M1 and M2; and Rho (meridian) angle (ρ), the angle between M2 and the plane Γ defined by the vector M1 and the normal to the plane formed by M1 and D. (**C**) Phylogenomic tree of structural domains reconstructing the evolutionary history of 3892 fold families (FFs) in 8127 proteomes sampling viruses and all major cellular taxonomical groups of the RefSeq database^31^. The evolutionary heat map describes the phylogenetic data matrix of genomic abundances derived using hidden Markov models of structural recognition^32^ used to build the tree using published methods^33^ in PAUP*^34^ with good performance^35^, with domains ordered according to their time of origin in a relative scale (*nd*) and rows describing the 8127-proteome set ordered according to a rooted tree of proteomes. FF abundance is described with a scale. Note biphasic abundance patterns in Eukarya, high diversity in Bacteria, homogeneity in Archaea, and sparse distributions in viruses. (**D**) A molecular clock of folds^36^ establishes that *nd* values of FFs domains were linearly corelated with geological time in billions of years (Gy) using Pearson (*r* = –0.974, *p* < 0.00001) and Spearman (ρ = –0.961, *p*(2-tailed) = 0). ρ < r rejects non-linear behavior. (**E**) Diagram illustrating an undirected bipartite EF network and its L and D projections unfolding along time events (t_1_, t_2_, t_3_ and t_4_). Nodes are described with symbols (circles = loops, rhomboids = domains), with size proportional to the number of links they establish.

We traced previously reported times of origin (evolutionary age) of domain structures^29^, recently used to trace multicellularity, translation, and ribosomal structures associated with protein folding^18^, over a bimodal graph-theoretic representation of domains and their associated loop prototypes. Later, we decomposed the bipartite representation into monomodal network projections^30^. Figure 1 illustrates the general strategy. The data pipeline involved the survey of domains and loops, their mapping to each other with bipartite networks, the assignment of times of origin from a chronology (series of time events) of domains, and the unfolding of a time series of networks describing recruitment patterns and evolution of an ‘elementary functionome’ (EF) of loop structures that are modular (Fig. 1A).

Loops were classified into prototypes using the exhaustive classification scheme of ArchDB^27^ based on structural geometry and conformation (Fig. 1B). Each ArchDB loop structure is a region of a PDB entry that associates with one ArchDB-classified loop prototype, which in turn makes up the structure of one or many domains. In our study, prototypes were identified using DS filtering of domains to loop mappings with e-value < 0.001^27^. They were named according to clustering method used (DS), ‘type’ of bracing secondary structures (HH, α-helix–α-helix; HE, α-helix–β-strand; EH, β-strand––α-helix; HG, α-helix–3_10_-helix; GH, 3_10_-helix–α-helix; GG, 3_10_-helix–3_10_-helix; EG, β-strand–3_10_-helix; GE, 3_10_-helix–β-strand; BN, β-β hairpin; and BK, β-β link), length of the aperiodic loop region between secondary structures, class (same conformation) and subclass (common geometry), in that order. For example, the ancient DS.HE.3.1.1 prototype present in ancient NAD(P)-binding Rossmann-fold domains has α-helix and β-strand bracing structures (HE), a 3 residue-long loop region, and the most populated conformation and geometry classes (ranked 1) (Fig. 1B). Structural domains defined at FF level were named using SCOP *concise classification strings* (*ccs*). For example, the tyrosine-dependent oxidoreductase FF that holds the ancient DS.HE.3.1.1 prototype has a *css* of c.2.1.2 typical of Rossmann folds. The ‘times of origin’ of domains were directly derived from a most parsimonious phylogenomic tree of domain structures generated from a census of domain abundance in 8127 proteomes (Fig. 1C). A heat diagram of the phylogenomic data matrix already reveals tantalizing abundance patterns differentiating the proteomes of Archaea, Bacteria, Eukarya, and viruses^29^. The highly unbalanced tree of domains permits to establish times of origin of FFs in a relative 0-to-1 scale of node distance units (*nd*). A molecular ‘clock of folds’ derived from calibration points of protein domain structures defined associated with microfossil, fossil and biogeochemical evidence [including molecular, physiological, paleontological, and geochemical markers and first appearance of clade-specific domains; first described in Wang et al.^36^] was used to convert relative *nd* ages of FF domains into geological time in Gy (Fig. 1C). As expected^37,38^, *nd* values of the most ancient FFs in fold superfamilies were strongly correlated with geological time (Fig. 1D). The chronology allowed to transfer the times of origin of domains to loops, imposing time directionality on network links (making them arcs with arrows pointing from older to younger nodes) and allowing construction of time series of networks that are growing in evolutionary time using methodologies developed by Aziz et al.^14^. Since ancestral loops are recruited into growing structures of domains to perform modern functions, their time of origin were borrowed from the most ancestral linked domains or from the second oldest domains when multiple domains shared loop structures.

The EF network is a bipartite graph with two segregate sets of vertices (nodes), one representing loops (circles) and the other domains (rhomboids) (Fig. 1E). Bipartite graphs can be decomposed into two one-mode projections using mathematical properties of finite graphs (Diestel^39^). These projections describe how one set of nodes interlink based on bipartite connections to nodes of the other set: links in the domain projection describe how domains share loops in their structural makeup, while conversely, links in the loop projection describe how loops combine to form structures around active sites in domains. Since loops host molecular functions^5^, and domains represent bona fide structural, functional and evolutionarily conserved units of proteins^28^, the time series of EF networks and their projections described how domains recruited molecular functions in protein evolution.

Evolving networks can be modeled using computer-based Discrete Event Simulation (DES) tools^40–42^. DES tools delineate the growth and behavior of complex networks as a sequence of discrete events, with time flowing from event to event as a *step function*. Here, we borrow the DES rationale by mapping the growing structure of the undirected and unweighted EF network and its directed weighted projections to evolutionary time intervals (Fig. 1E). This is further illustrated with a toy example in Supplementary Fig. S1.

### A frustrated and ongoing history of modular and non-modular loop recruitment

**Figure 2:**
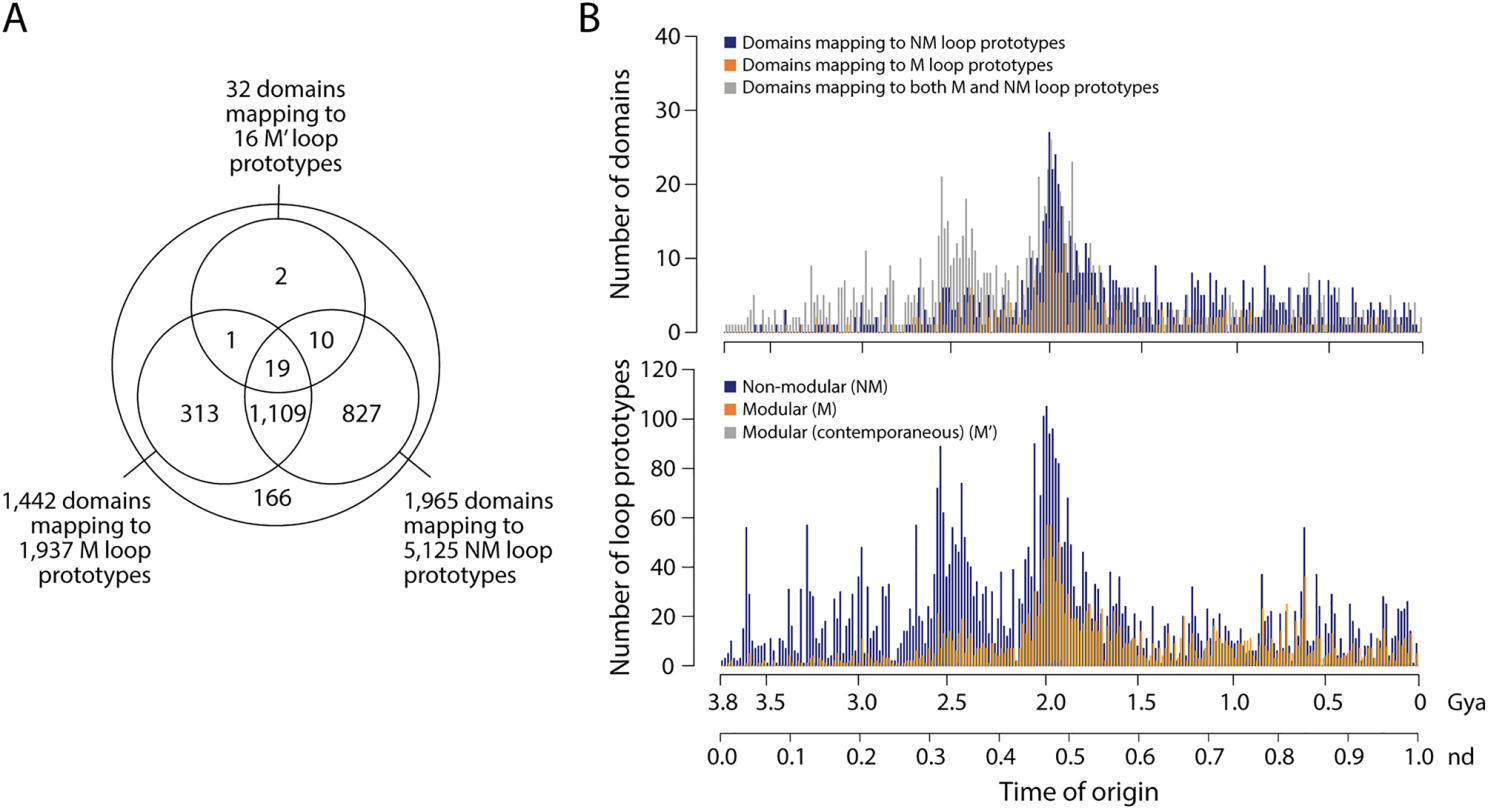
Mapping loop prototypes to domains along the domain chronology. (**A**) Venn diagram describing how structural domains map to modular (M and M’) and non-modular (NM) loop prototypes. The set of 166 domains does not map to loops but can be traced to a set of 228 filtered M loops. (**B**) Tracing domains and loops along the evolutionary timeline. The gradual appearance of domains belonging to the three most populated groups of the Venn diagram along domain history reveals the early rise of domains mapping to both M and NM loops in evolution. The gradual appearance of modular and non-modular loop prototypes reveals the early rise of NM loops in evolution.

We clustered 88,321 ArchDB loop structures mapped to 3892 domain families of our phylogenomic timeline, filtered at an e-value of < 0.001 to minimize false positives while detecting reliable structural and functional associations at statistically significant levels. This clustering yielded 7078 loop prototypes with 9650 many-to-many mappings to 2447 domains. The 7078 loop prototypes were divided into three subsets according to how they associated with domains across the evolutionary timeline (Fig. 2A). A subset of 5125 *‘non-modular’* (NM) prototypes mapped uniquely to individual domains of a same age belonging to a set of 1965 domains. In contrast, a subset of 1937 *‘modular’* (M) prototypes mapped to more than one domain out of 1442 domains with times of origin spread throughout the timeline, with 2546 mappings. These prototypes acted as modular units of structural, functional and evolutionary significance. They represented the most abundant, widely distributed, and interesting loops of this study. Finally, a small subset of 16 *‘modular’* (contemporaneous) (M’) prototypes involved associations with more than one domain that occurred within individual time events. While we define modules as sets of integrated (coordinated) parts that cooperate to perform a task and interact more extensively with each other than with other parts and modules of a system, we recognize modules by the property of ‘modularity’, the degree to which parts of a system can be separated and rearranged in different contexts.

A Venn diagram of domains mapping to the three groups of prototypes revealed three Venn groups of especial interest, domains mapping to only M loops (313 domains), domains mapping to only NM loops (827), and domains mapping to both M and NM loops (1109) (Fig. 2A). The fact that a significant number of domains are recruiting both M and NM loops suggest their makeup is shaped by two recruitment mechanisms, one stochastic and the other evolutionary. It is likely that NM loops are stochastically drawn into domains by local genome rearrangement activities but are never coopted by other domains because they fail to add significant protein functionalities. In contrast, M and M′ loops behave as true evolutionary units capable of distributing structural and functional novelties to many domains along the timeline. Their evolutionary recruitment enables a combinatorial origami of modular structural motifs benefiting protein evolution. Figure 2B traces the three Venn groups of domains and the three groups of loop prototypes along the evolutionary chronology. Remarkably, domains mapping to both M and NM loops accumulated earlier than domains mapping only to either M or NM domains along the chronology. In addition, the presumed more stochastic NM loops also accumulated earlier than the M and M’ loops in evolution. Our observations are compatible with highly dynamic views of protein organization (e.g.,^43,44^) or the existence of molecular discriminating Maxwell demons that dissipate energy and information^45^. These views foster molecular systems that are frustrated by ‘messiness’ in the form of stochastic noise, heterogeneity, infidelity, and variation, as nicely exemplified by the existence of intrinsic disorder embedded in the structural makeup of proteins.

### Time event ‘waterfall’ networks uncover the birth of domains in protein evolution

**Figure 3:**
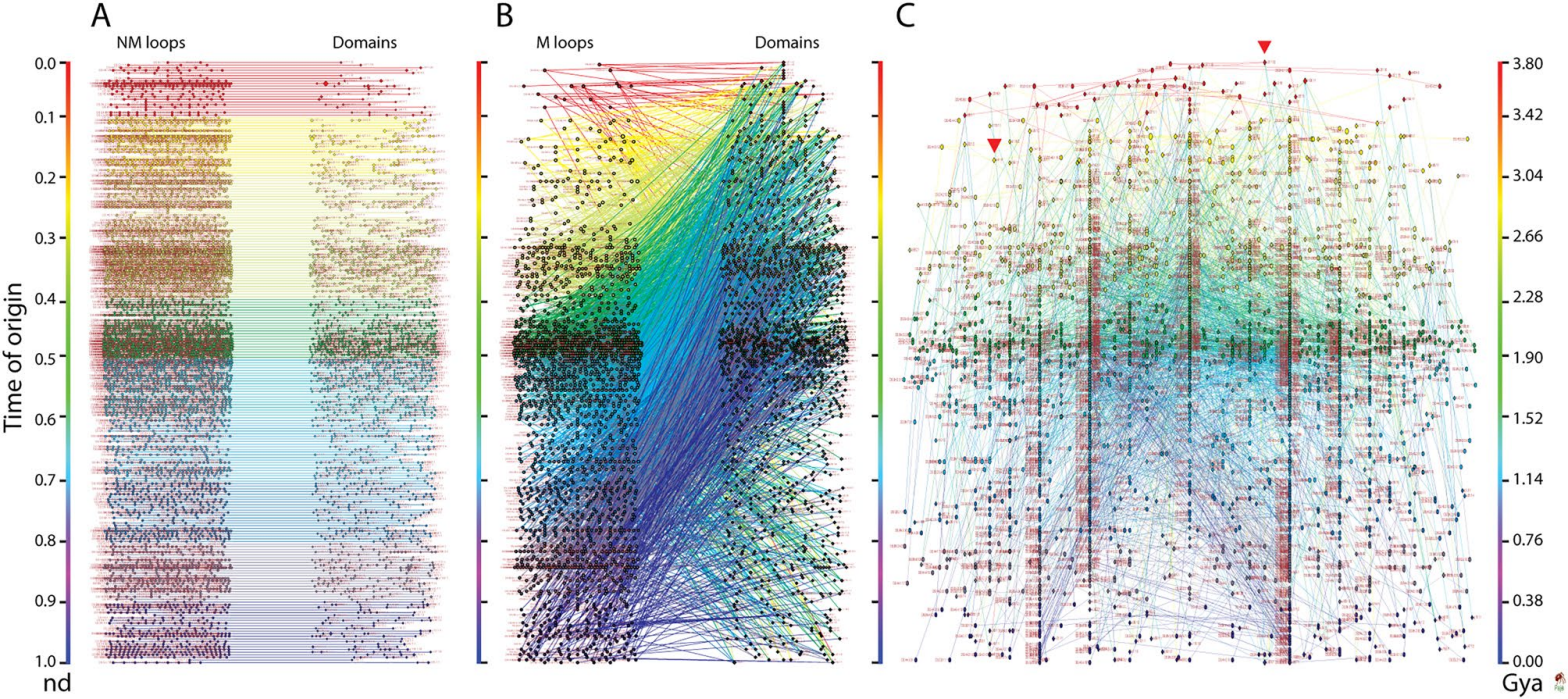
Bipartite networks describe the origin and evolution of structural domains by recruitment of non-modular (NM) and modular (M) loop prototypes in protein evolution. Networks uncover how domains share NM prototypes (**A**) or M prototypes (**B** and **C**) along the evolutionary timeline. Loop and domain nodes are colored according to time events, labeled using established ArchDB and SCOP nomenclature, respectively, and arranged top-down according to time of origin (age, *nd*) displayed on a relative 0-to-1 scale or on a ‘billions of years ago’ (Gya) scale time-calibrated with a molecular clock of domains. The network linking M prototypes to domains in bipartite (**B**) and time event waterfall format (**C**) represents an evolving ‘elementary functionome’ (EF) describing the recruitment of protein loop modules. Nodes were scaled proportional to their weighted degree, i.e. the sum of the weights of all edges of the nodes. Prototype hits to structural domains in proteomes were not used to weight edges to avoid complication in interpretation of weighted network projections. Red arrowheads indicate the origin of major waves of recruitment in the waterfall network. The horizontal expansion is dictated by VOS clustering, which elucidates formation of modules along the evolutionary timeline (see methods).

To further dissect the different recruitment strategies, we constructed two bipartite networks, one describing the evolutionary recruitment of NM loops and the other describing the recruitment of M loops into the structure of domains. The evolving bipartite network that links 5125 NM loops to 1965 domains uncovered ‘horizontal’ recruitments occurring throughout protein evolution, always restricted to individual time events (Fig. 3A). Note that meaningful monomodal projections of this network cannot be produced because there are no links other than the horizontal single loop-to-domain mappings. A multitude of ‘temporal’ recruitment waves in the form of ‘ripples’ were however evident throughout the timeline, with at least 5 significant ripples occurring between 3.8 and 2.7 Gy ago (Gya) and two subsequent (major and distinctive) ripples occurring ~ 2.5 Gya and ~ 2 Gya. This series of small waves embody a multiplicity of subnetworks unfolding in time, which is in sharp contrast with waves that involve individual subnetworks with single origins, which we will later discuss. The recurrent patterns of the waves in these ripples clearly show that the stochastic mechanisms of cooption we propose are ongoing. In sharp contrast, the evolving bipartite network that links M loops and domains shows ‘vertical’ recruitments occurring between the 1937 modular loop prototypes and 1442 domains throughout the timeline (Fig. 3B). Visual inspection of the network showed waves of co-option, some of which matched the major ~ 2.5 Gya and ~ 2 Gya ripples of the non-modular network and involved pervasive recruitment of older loops. This network is the most significant because it explains how loop recruitments have shaped the structure of domains in the protein world. It is truly an EF network, which can be fully dissected into loop and domain projections. Given its evolutionary centrality, our focus will shift to this network.

**Figure 4:**
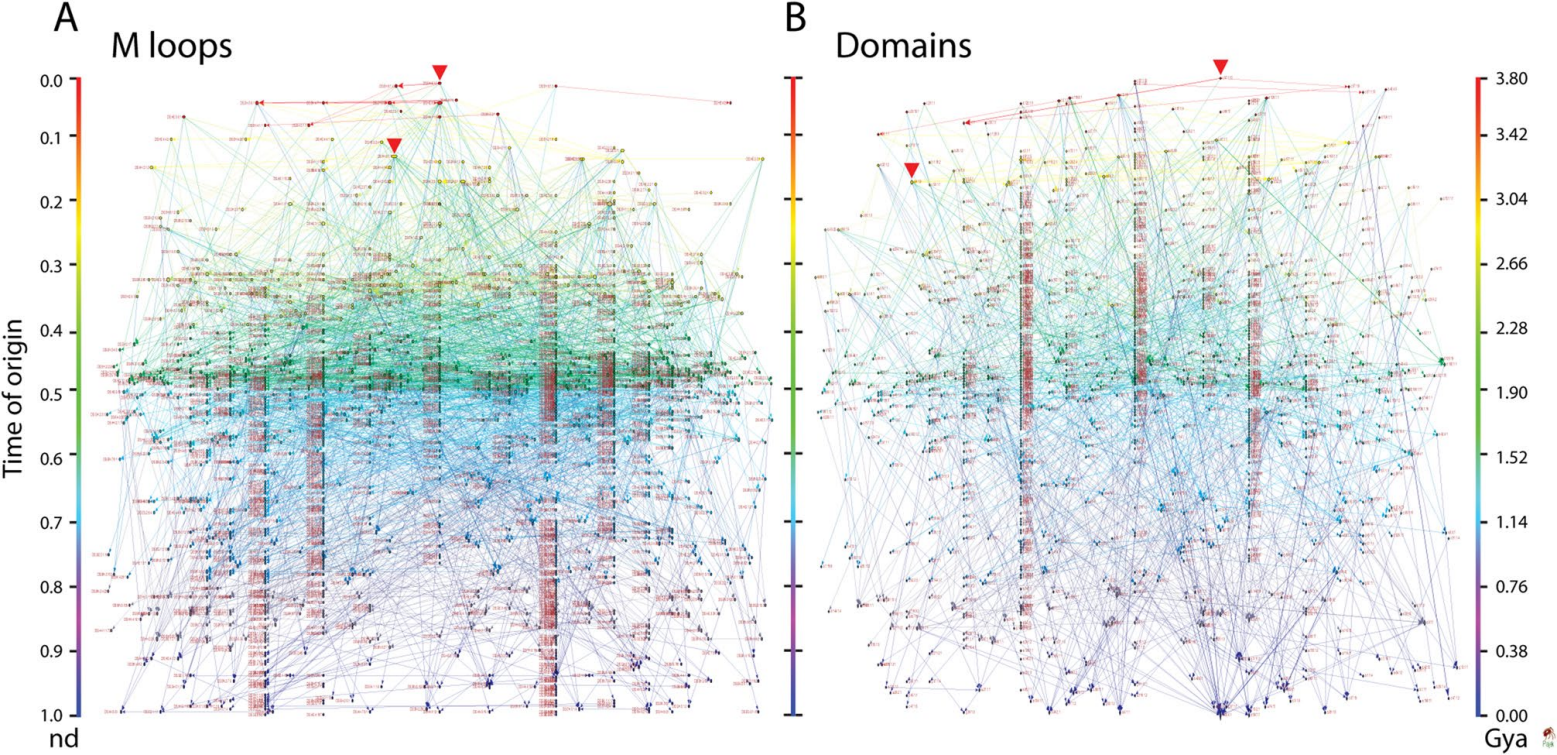
EF network projections in waterfall layout. (**A**) Loop network defined by 1937 prototypes (ellipsoids) and arc connections (arrows) representing sharing of domain structures. (**B**) Domain network defined by 1442 domains (rhomboids) and arc connections representing sharing of loop prototypes. Loop and domain nodes in their uni-modal graph representations were labeled using established ArchDB and SCOP nomenclature, respectively, arranged top-down in the order of time events, and colored according to age (*nd*) on a relative 0-to-1 scale or on a ‘billions of years ago’ (Gya) scale. Links (directed) were colored by the age of a destination node.The 2-dimensional scale of nodes was kept proportional to their weighted degree. In particular, the horizontal and vertical sizes of the loop and domain symbols were made proportional to the weighted outdegree and indegree, respectively, showcasing source-and-sink relationships. All weighted degree vectors were shifted by a value of 10 to avoid vanishing of 0-degree entities. The width of arcs joining the loops and domains was made proportional to the number of shared domains and loops, respectively. Red arrowheads indicate the origin of major waves of recruitment in the time event waterfall. The arcs symbolize the flow of time (random direction for contemporary nodes).

The bipartite EF network of Fig. 3B embodies an undirected graph of 2546 links with a *network density* (actual**/**possible number of links) of 0.0009 [2546/(1442 × 1937)] and a *node average degree* (links per node) of 1.507 (± 0.018), i.e. loop components had approximately less than 2 interlinks on average. Visual inspection revealed that the network was well clustered. The Visualization of Similarity (VOS) clustering method^46,47^ uncovered 889 communities (also known as modules) with a high *modularity index* of 0.996. The adaptive events of the EF network were made vivid by color coding and arranging the component nodes by age in a top-down bimodal layout that followed the evolutionary timeline of domain structures (Fig. 3B). The node sizes were made proportional to node connectivity, measured by weighted degree, highlighting the hub-like behavior of the network structure. In order to better visualize evolutionary network patterns, the VOS clusters (comprising of hubs and their neighbors) were spread horizontally using the energy-optimized Kamada-Kawai^48^ ‘free’ and ‘optimize inside clusters only’ methods (Fig. 3C). The resulting ‘waterfall’ network layouts vividly illustrated functional recruitment responsible for how loop modules make up domains along the events of the entire evolutionary timeline. Consequently, EF network projections were also visualized in waterfall layouts (Fig. 4). The connectivity of these monomodal networks was made evolutionarily explicit by giving a direction to the intra connecting nodes with *arcs*. The resulting loop and domain directed networks had 2024 and 1005 arcs each, with *network densities* of 0.00054 [2024/(1937 × (1937 − 1))] and 0.00048 [1005/(1442 × (1442 − 1))] and total *node average degrees* of 2.104 (± 0.061) and 1.415 (± 0.067). In other words, ~ 2 domains and ~ 1.5 loops were shared on average, respectively. Both the loop and domain monomodal networks also showed significantly high community structure with 879 and 882 clusters each, and modularity indices of 0.994 and 0.990, respectively. The number of outward (outdegree) and inward (indegree) directed links (arcs) defined nodes as ‘donors’ (sources) or ‘acceptors’ (sinks), respectively. The horizontal and vertical scale of node symbols were made proportional to the weighted outdegree and indegree, respectively. This made the visualization of hubs explicit, e.g., a transition from wide to tall symbols along events indicated source-sink morphing dynamics. Overall, these transitions expressed an expected increase in the probability of co-opting older loops and domains with time, but also a surprisingly continual recruitment process operating among recent molecular forms.

### The evolutionary emergence of scale-free properties in the EF network

**Figure 5:**
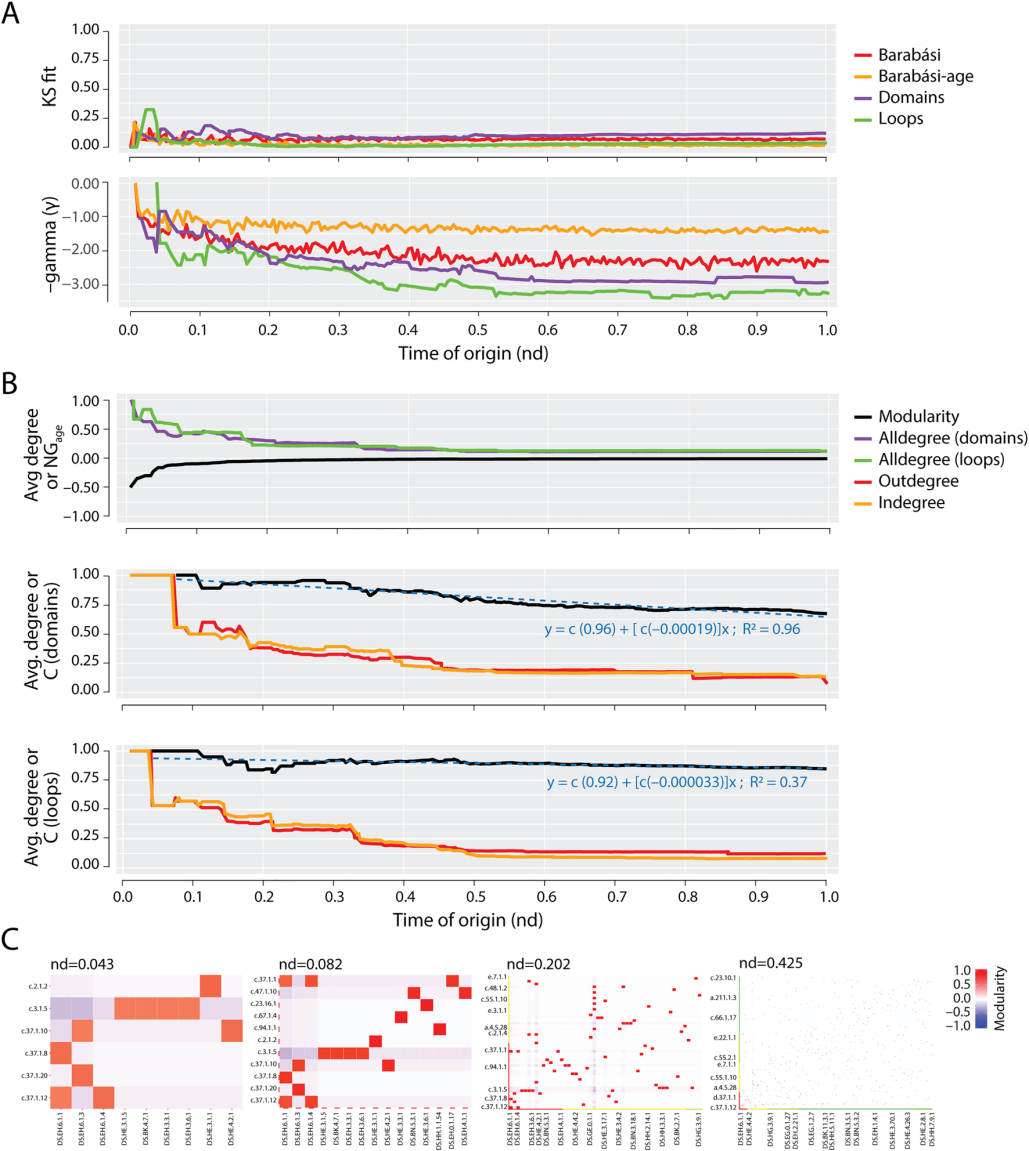
Emergence of scale-free and modular behavior in the evolving EF network. (**A**) Transfer of the scale-free property in the EF network. The KS fit statistic measures network degree deviations from the fitted power law distribution. Lower KS values indicate better fit. The reference Barabási (red) and Barabási-age (orange) curves are included for comparison. The generated scale-free network controls consider the preferential attachment probability of an old node to be proportional to its degree (Barabási) or to both its age and degree (Barabási-age). Power law decay γ exponents of the lower panel ‘scale-free’ levels of heterogeneity in networks, with γ > 2 describing typical heavy-tailed distribution of connectivity. (**B**) Modularity of growing networks. *NG* with default membership (partition) defined by age (*NG*_*age*_) was computed for the EF network. *NG*_*age*_ indicates mixing of nodes by age in an assortative (≥ 0) or disassortative (< 0) manner across modules^51^. The average *Clustering Coefficient* (*C*) for loop and domain networks describes the averaged ratio of the triangles to the connected triples over all nodes, where the networks are simplified (undirected/unweighted)^52–54^. We report the coefficients of linear regression models (blue lines) over *C* for the domain network as − 0.00019 by network size (*N*) and − 0.350 by age, and those for the loop network as − 0.000033 by *N* and − 0.091 by age. Linear regression lines shown are by N. Normalized average degree (avg. degree) curves, computed as mean-/ max-degree of the network at an event, were included as reference controls. Separate curves were computed for the ‘alldegree’ of loop and domain portions of the EF network and for the ‘outdegree’ and ‘indegree’ of loop and domain networks. Degrees were cumulative and weighted. Scores and indices were calculated for each event of the evolving networks. Time of origin (*nd*) is indicated in a relative 0-to-1 scale. (**C**) Progression of pairwise modularity in the EF network. The cells of the heatmaps represent modular strength between a loop and domain as compared to their individual connectivity with the rest of the network, scaled by the absolute log10 value of the network wide modularity index *NG*_*age*_ at that event^51^. The first three panels illustrate the hidden switch of power law and modularity properties between loops and domains. The last panel corresponds to the most-distinguishable plauteued EF network. The significant loop prototypes and domain structures involved in the two major waves of functional innovation along with sister hubs having red tiles are displayed using established ArchDB and SCOP nomenclature, ordered ascendingly and color-coded according to node age.

When nodes of a growing network draw links in a ‘rich-get-richer’ manner, the network follows a preferential attachment model in which the probability *P(k)* of a node linked to *k* nodes decays as a power law, *P(k)* ~ *k*^–γ^, and does so without a characteristic scale. These scale-free networks are ubiquitous in biology, and in general have regression exponents γ = 2.1–2.4 with typical heavy-tailed distributions^49^. For metabolic networks of organisms in all superkingdoms, γ = 2.2 (e.g.,^50^). We tested if the evolving EF network and its projections were scale free by studying their degree distributions along the time series of the growing networks (Fig. 5A and Supplementary Fig. S2). Remarkably, analysis of the cumulative connectivity (of links or arcs) with appropriate statistics revealed that a power law tendency, along with associated ‘scale free’ generative models, was an emergent property in the EF network, but not in its projections.

Several statistics could not reject power law connectivity behavior in the most ancient loops and domains very early in evolution (*nd* ~ 0–0.02), domains but not of loops during the *nd* ~ 0.02–0.04 interval, then of loops but not domains during *nd* ~ 0.04–0.07, and finally of loops but not domains of the EF bipartite network throughout the rest of the timeline (Fig. 5A and Supplementary Fig. S2). Failure to reject was evaluated with the Kolmogorov–Smirnov (KS) statistical test of power law fit^55,56^ (Fig. 5A). High *p*-values of the KS test (≥ 0.05) and low values of the KS fit statistic (≤ 0.10) failed to reject a fitted power-law distribution. Fitting the power law distribution produces decay exponent α. Values of α higher than 1 supported assumption of probability of power law fit *P*(X = x^–α^) for example for later degree distributions of loop components of the growing EF network. However, the log-likelihoods of the fitted power law gradually deviated towards larger negative values for most network events of the timeline, diminishing the likelihood of power law distributions. Analyses of the growing EF networks therefore reveal remarkable patterns of power law emergence and transfer. While power law behavior was shared between the early-evolved loop component and the domain component of the bipartite network, these ancient components went through two cycles of an exchange of scale-free properties, from domains to loops and then from loops to domains, as molecular functions developed in protein evolution (Fig. 5A; Supplementary Fig. S2). Log-linear regression models overlapping the power law curves showed that the coefficient of power law decay γ followed the scale-free cycles but with a pervasive tendency to increase in evolution (Fig. 5A). Beginning from a linear scale (γ = 1.000, *nd* ~ 0.01), γ increased through fluctuations evident well before the first ripple occurring ~ 2.4 Gya (mentioned above), reaching a very strong power law scale for the loop portion (average γ = 2.877 ± 0.046 from nd ~ 0.33 onwards, with max γ = 3.450 at *nd* ~ 0.85) and domain portion (average γ = 2.456 ± 0.034 from nd ~ 0.31 onwards, with max γ = 2.915 at *nd* ~ 0.84) of the EF network, with coefficient of determination (R^2^) of ~ 95% supporting the linear models. Similarly, the loop and domain network projections maintained strong (average γ = 2.270 ± 0.030) and moderate (γ = 1.865 ± 0.038) power law scale, respectively. Thus, the extent of preferential attachment of our recruitment networks is in general more robust than that reported for metabolic networks^52^. The extraordinary observation of a dual ‘yin-and-yang’-like power law transfer between loop and domain components may be indicative of a continuing global scaling phenomenon in a biphasic emergence of biological modules^57^, which we now explain.

### The rise of hierarchical modularity

Networks become modular when their nodes connect to each other within bounds of a community (module)^51^. Modularity offsets scale-freeness by balancing the degree distribution of nodes in the modules of the networks^50,58^. However, these opposing properties reconcile when modules are integrated hierarchically^52^. A primary measurement of modularity is the *average clustering coefficient* (*C*), a ratio of triangles (graph cycles of length 3) to connected triads in the network, averaged over all nodes, while ignoring edge directionality and weights^52,53^. Since *C* for the bipartite EF network was not meaningful due to absence of triangles, modular organization was investigated through its projections (Fig. 5B and Supplementary Fig. S3). The domain and loop networks exhibit *C* values of ~ 0.805 (± 0.0066) and ~ 0.893 (± 0.0025), respectively, significantly higher than ~ 0.6 reported for metabolic networks^52,58,59^. The elevated *C* of EF network projections suggests integration of modules of loop prototypes and domain structures, which are densely connected, by few sparsely connected links between them. Thus, the EF network has a highly cohesive structure of modules.

A notable property of *C* is its sharp decline with network size N for scale-free models^60^, as N^−0.75^, contrary to highly modular networks that are independent of N (e.g.^52^). For the domain and loop networks, *C* regressed with N as N^–0.00019^ and N^–0.000033^, and with age *nd* of the networks as *nd*^–0.35^ and *nd*^–0.091^, respectively (Fig. 5B and Supplementary Fig. S3), confirming the modular structure of the evolving networks. The smaller exponents suggest the increased ‘granularity’ of the modular makeup of the loop network compared to that of the domain network, supporting earlier observations that lower levels of organization in bipartite networks of metabolism were more granular and cohesive^61^. Expectedly, ‘Barabási’ reference controls strictly following power-law had *C* = *0*^62^. Evolving domain and loop networks showed trends of modularity and scale-free properties were anticorrelated. For example, the *C* of domain and loop networks showed two initial cycles of fall and rise in modularity (a drop from 1.000 to ~ 0.889 and ~ 0.950 at *nd* ~ 0.112, rise to ~ 0.956 and ~ 0.894 at *nd* ~ 0.258, and then a dip to ~ 0.830 and ~ 0.892 at *nd* ~ 0.356, respectively), followed by a plateau to ~ 0.73 and ~ 0.88 at *nd* ~ 0.635, respectively. These patterns matched the power law trends, as indicated by KS fit indegree statistic, which manifested slightly earlier than the corresponding modularity phases and rejected power law behavior in initial phases (a rise from 0.000 to ~ 0.2 each at *nd* ~ 0.077, fall to ~ 0.1 each at *nd* ~ 0.146, and then a peak to ~ 0.2 and ~ 0.12 at *nd* ~ 0.202, respectively), before plateauing to ~ 0.06 and ~ 0.1 at *nd* ~ 0.339, respectively (Fig. 5B; Supplementary Figs. S2 and S3). These counteracting trends of modularity preceding scale-free behavior with lags of ∆*nd* ~ [0.035, 0.112, 0.154, 0.296] in the domain and loop networks likely reflect transfer of scale-free properties from loops to domains of the EF network and generative cycles of modular and hierarchical network structure.

To test this notable conjecture, we studied three measures of modularity along evolving networks, the Newman-Girvan (*NG*) index partitioned either by age (*NG*_*age*_) and by VOS (*NG*_*vos*_) and the Fast Greedy Community (*FGC*) index. The *NG* algorithm calculates the maximum number of shortest paths running through an edge, a property known as ‘edge betweenness’^51^. The algorithm detects communities (modules) by progressively removing edges with high betweenness in iterative fashion. *NG*_*age*_ ranges from –1 to 1, with positive values indicating modular structure within age events, while negative values indicating otherwise. *NG* partitioned by VOS (*NG*_*vos*_) describes VOS membership cohesiveness^46,47^. The *FGC* detection algorithm uses a hierarchical agglomerative approach of iteratively sampling random links that would increase the modularity of an initial subnetwork linking highly connected nodes in the original network^63^. Remarkably, the *NG*_*vos*_ and *FGC* indicators measured along the timeline uncovered mirrored patterns of increase in modular cohesiveness and agglomerative structure for all growing networks. Conversely, *NG*_*age*_ indicated divergent progress towards age-associativity in the EF network and its projections. All three networks showed age-independent origins with *NG*_*age*_ ≤ − 0.3, − 0.25 and − 0.5 until *nd* ~ 0.034, ~ 0.073 and ~ 0.039, respectively. Loop and domain networks then parsimoniously progressed towards age-associativity with an early rise of *NG*_*age*_ to ~ − 0.2 and then to ~ − 0.1 at *nd* ~ 0.039 and ~ 0.056, respectively, in the EF network, and at *nd* ~ 0.077 and ~ 0.112, respectively, in the domain projection, before plateauing out to ~ − 0.01 at nd ~ 0.545 in EF and to ~ − 0.006 at nd ~ 0.575 in the domain network. However, the loop projection became aggressively age-associative early in evolution, with an initial increase of *NG*_*age*_ from − 0.5 to ~ − 0.07 at *nd* ~ 0.43 followed by a gradual rise to ~ 0.29 at *nd* ~ 0.18, before plateauing out to ~ 0.02 at nd ~ 0.528 (Fig. 5B; Supplementary Fig. S3). These network modularity patterns are indicative of more robust age-wise cohesive recruitment in loops than domain structures. This trend of course approached an equilibrium as network agglomerative modularity matured and emerging structures were widely recruited throughout the timeline. This recruitment trend was also evident in the pairwise *NG*_*age*_ heat maps of the EF network (Fig. 5C; Video 1): a red sigmoidal signal during early events (first three panels) diffused into a red pixelated pattern These modular matrix representations along with power law and modularity statistics reflect the clustering of modules into modules typical of hierarchical modularity matching the clustered scale-free organization of metabolic network^52^ (Supplementary Fig. S4; Video 2). In contrast, recruitment initially drove the growth of loop and domain networks, but its impact was counteracted by age-bound modularity. Thus, our network timelines revealed a hidden switch to hierarchical modularity that transferred scale-free properties between loop and domain structures ~ 3.4 Gya. The timing of this switch, as discovered earlier in the literature^14^, overlaps with the early development of genetic code specificity in emerging aminoacyl-tRNA synthetases and the ribosome, overall enabled by the OB-fold structure^1^ (Fig. 2).

The evolutionary rise of scale-freeness and hierarchical modularity in the emerging EF network of loop prototypes and domain structures is a prediction of the biphasic (bow-tie) theory of module emergence proposed by Mittenthal et al.^57^ to explain concurrent patterns of unification and diversification existing in biological systems. In a first phase, the nodes of the emerging network associate variously, but with weak linkages, through processes of recruitment. As the system grows, nodes diversify by competitive optimization of enhanced functionality. Useful emerging interactions constrain node associations, causing tight linkages to self-organize into tightly associated communities. In a relatively longer second phase, variants of these modules evolve and instigate a new generative cycle of higher-level organization, highlighted by scale-free module recruitment. The network paradigm formalizes the concept of ‘linkage’ by using nodes to represent parts of the system and using links to represent their interaction and/or association. Biphasic patterns exist in dipeptide makeup, loop flexibility, and size of proteins^1,26^. Such patterns were also evident in several biological networks with dynamics unfolding at different time scales, from nanosecond dynamics to billions of years of evolution. For example, we recently uncovered biphasic patterns in evolution of domain organization^64^. The EF network now showcases its biphasic structuring by integrating communities of interacting structural parts of domains into modular classes of molecular functions. Thus, adaptations to a biphasic pattern of change appear to be a general biological phenomenon.

### Untangling patterns of molecular innovation and reuse of structures and functions

The waterfall EF, domain and loop network layouts arranged unique time events (228, 226 and 206, respectively) along a timeline that spans from the origin of proteins (*nd* = 0) to the present (*nd* = 1) (Figs. 3 and 4). An analysis of how nodes connect to each other across these events dissects the combinatorial recruitment process that embeds loop prototypes into domain scaffolds to generate new molecular functions. Crisscross patterns in network links strongly suggest recruitment of old loops by younger domains throughout the timeline. In fact, the largest hubs holding most of loop and domain connectivity were observed appearing very early in protein evolution, drawing heavily from innovations appearing during the first 800 million years, but then rigorously extending recruitment from ~ 2.5 to ~ 1.25 Gya of protein history (Figs. 3 and 4). This confirms the proposal that loops that are most abundant and widely distributed in genomes are likely the oldest^10^.

**Figure 6:**
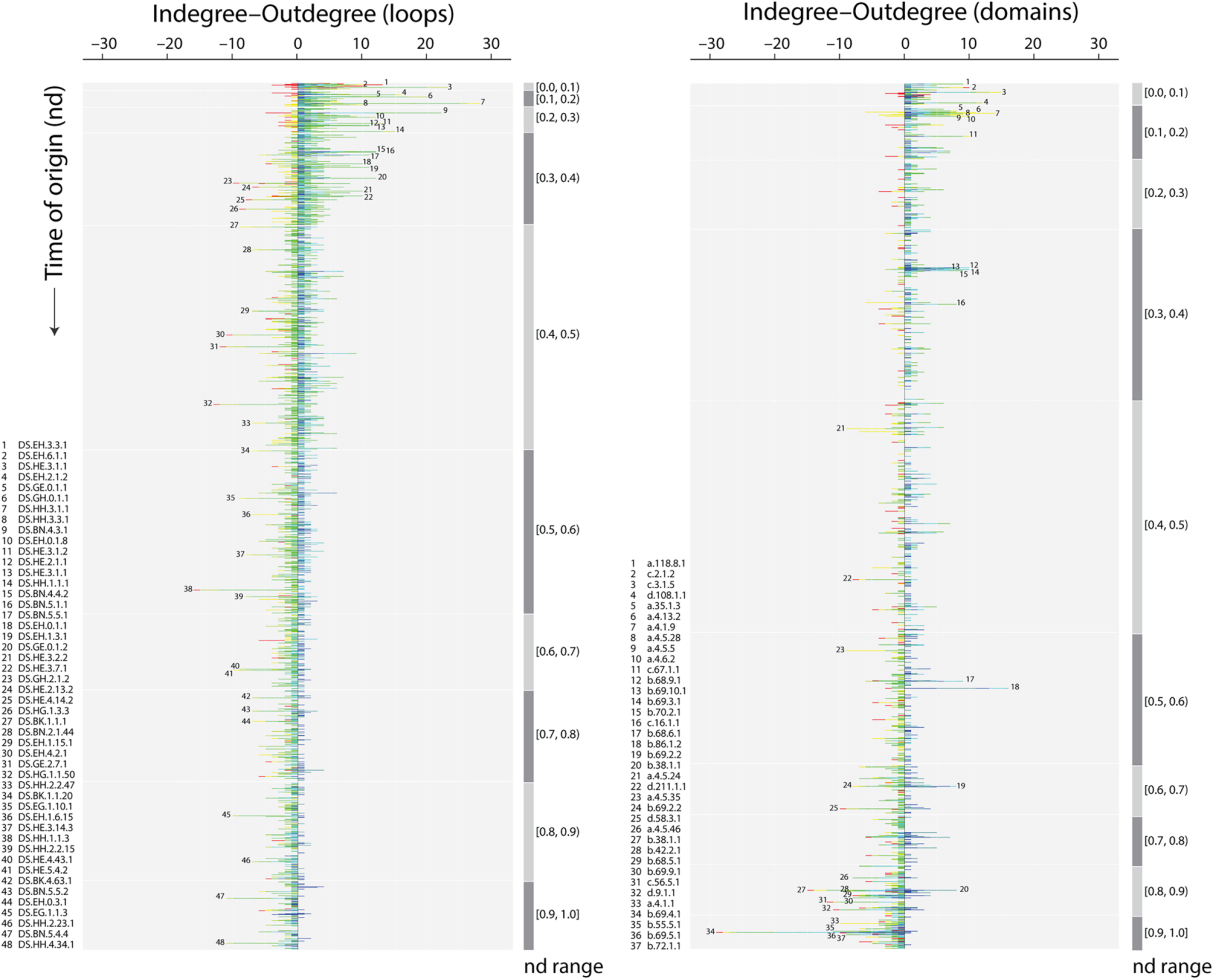
Chronological accumulation of connectivity in loop and domain networks. The stacked bar charts depict the chronological accumulation of connections (arcs) in the loop and domain networks along time events of the timeline. Nodes in 99th percentile of connectivity are labeled using ArchDB and SCOP nomenclature. Each event corresponds to the discovery of loops and domains from one of 206 and 226 events, respectively, along a timeline that spans the origin of proteins (*nd* = 0) and the present (*nd* = 1). For visualization purposes, the timeline of events was coarse-grained into 10 age bins. For each node, the number of connections to nodes appearing earlier (indegree) or later (outdegree) in evolution were recorded and displayed as colored stacks in the stacked bars colored red-to-blue following time. The charts portray sink-source relationships in the recruitment of elementary functions viewed from the perspectives of loops and domains.

**Figure 7:**
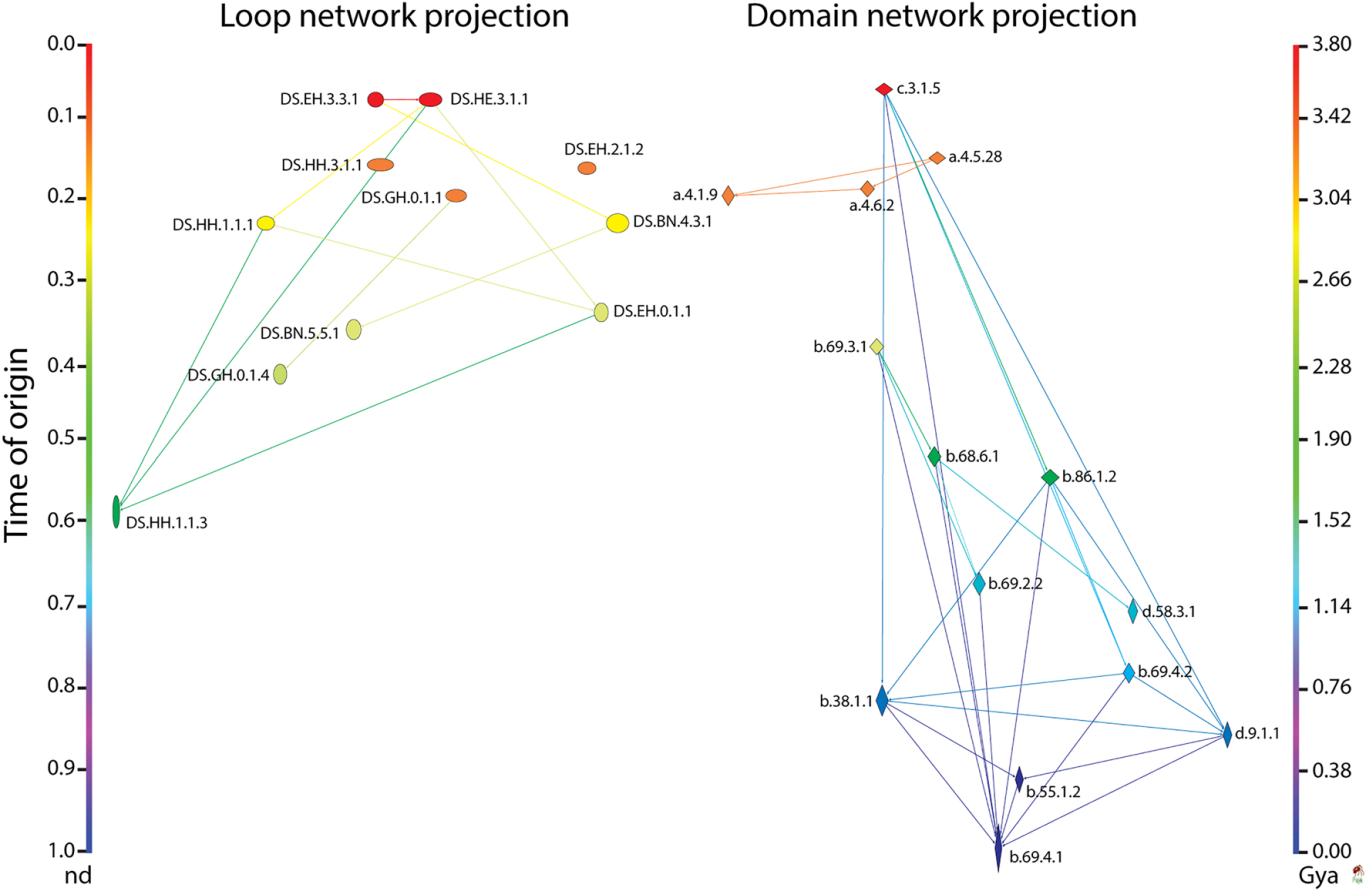
EF network projections in waterfall layout describing the evolution of loops and domains with the largest (100th percentile) network connectivity. The loop and domain network projections of 1442 and 1937 nodes, respectively, were each reduced with the restrictive criterion of excluding nodes with combined outdegrees and indegrees ≤ 99% of those of the rest of the nodes. The set of arcs (arched arrows symbolizing flow of time) in each network was also reduced to pairing events between nodes with 100th percentile connectivity and excluded those between contemporary nodes. Nodes are arranged top-down and colored according to age (*nd*) on a relative 0-to-1 scale or on a ‘billions of years ago’ (Gya) scale that describes evolutionary time events. Arcs are color-coded according to the age of the impending or more recent of the joined nodes. Loop and domain nodes were labeled with ArchDB and SCOP descriptors, respectively. To showcase source-and-sink relationships, node symbol sizes were scaled proportional to the weighted outdegree and indegree along the horizontal and vertical axes, respectively. Weighted degrees were shifted by a value of 10 to include 0-degree nodes for better visualization. The modular spread of nodes was based on VOS clustering.

While contemporary co-option of ancient loops and domains was prominent at every time event, most events of recruitment involved loop acceptors originating at *nd* = [0.3–0.8) and domain acceptors originating at *nd* = [0.4–1.0] (Fig. 4). Bar plots describing the accumulation of links in network evolution dissected both source-sink relationships and evolutionary span of network connectivity (Fig. 6). The plots demonstrated an overwhelming majority of modern recruitment events, some very recent, with relatively younger sink nodes (*nd* = (0.5–1.0]) being acceptors of very old donors or source nodes originating at *nd* < 0.3. In this respect, sink loops seem to be particularly adaptive, progressively drawing innovation from donors spanning the entire timeline. Box-and-whisker plots of cumulative weighted indegree and outdegree across network chronology (Supplementary Fig. S5) and scatter plots with linear distribution models of degree totals at *nd* = 1 (Supplementary Fig. S6) provided further insight into the patterns of contraction and expansion of mutually adaptive loop and domain innovations. Specifically, individual domains took advantage of the repertoire of very ancient donors for their functional tasks and showed signs of co-option among modern domains late in evolution (nd ≥ 0.8), supporting evolutionary patterns of recruitment observed in metabolic networks^65^. Similar patterns were identified when exploring the evolution of protein domain organization^64^ and EFL-mediated elementary functions^14^. In these studies, most innovations happened during the first ~ 1.8 Gy of protein history (Fig. 6). Analysis of the highly connected loop and domain subnetworks of the EF network projections showed that although the largest hubs appeared early in evolution, recruitment was generative of new hubs throughout the modern structural world (Fig. 7, Videos 3 and 4).

Finally, the connectivity of loop and domain components of the EF network gradually evolved from 1 to a global average of 1.77 (± 0.032) loops per domain and 1.31 (± 0.018) domains per loop, respectively (Supplementary Fig. S7). Remarkably, loop connectivity fluctuated up (~ 1.7 domains per loop), down (~ 1.2) and up again (~ 1.3) during early protein evolution (*nd* < 0.2). Domain connectivity fluctuated in a mirror fashion but with a slight phase delay (∆*nd* = 0.02) and with peaks of ~ 1.8, ~ 1.5 and ~ 1.7 loops per domain. These dual hourglass trends suggest a frustrated dynamics of growth in the number of loops making up the active sites of structural domains.

The connectivity patterns we identified exposed a fluid emergence of functional loops and domain structures in protein evolution. Their adaptive formation occurred at different rates in ripples and waves of recruitment and innovation. While the early appearance of loops provided raw materials for loop combinatorics throughout protein history, the ongoing introduction of loop structures and their repeated combination with older domains suggests that old loops are evolvable forms that are still evolutionarily active instead of relics headed for extinction. Remarkably, phylogenetic studies demonstrate similar dynamics materializing with domain structures and their combinatorial use in multidomain proteins^22,65^.

### Two primordial recruitment waves

Two primordial waves of functional innovation arising from ancient ‘*p*-loop’ and ‘winged-helix’ domains were originally identified in metabolic pathways^66^. Later evolutionary studies of elementary functionome^14^ and protein domain organization^64^ also uncovered these same two waves of innovation. Remarkably, the waterfall diagrams of the modular EF network and its projections (Figs. 3 and 4) revealed that these ancient recruitment pathways arising from ‘*p*-loop’ and ‘winged-helix’ domains were also presents in our networks, uncovering separate origins of sandwich, barrel and bundle domain structures. The versatility of the waterfall visualization in the form of highly connected (reduced) subnetworks of the hubs in the loop and domain projections visually untangled the two recruitment waves (Fig. 7). The realization that these evolutionary patterns are parallelly uncovered with various data sources, as depicted by simulated movies as well (Videos 5, 6 and 7), is remarkable, and strongly supports the historical statements we here elaborate.

The first larger wave originated in the *p*-loop containing nucleoside triphosphate (NTP) hydrolase domains (c.37.1.12, c.37.1.8 and c.37.1.1) (Figs. 4A, 7A) and their contemporary and relatively long *p*-loop-related DS.EH.6.1.1 and DS.EH.6.1.4 prototypes, with eight relatively recent terminal loop prototypes, DS.EH.0.1.17, DS.EH.6.1.2, DS.HE.3.70.1, DS.EH.2.17.1; DS.BN.2.3.4; DS.GH.2.2.2, DS.HE.0.1.1 and DS.GH.2.1.4, respectively (Figs. 4B, 7B). These domain families of the P-loop containing nucleoside triphosphate hydrolase (c.37.1) superfamily are the most ancient and most popular Rossmanoid α/β/α-layered domain structures of a chronology of domain history^19,20,37,66^. The *p*-loop prototype of the *p*-loop hydrolase fold enabled nucleotide triphosphate binding functions mediated by the Walker A (*p*-loop) sequence motif, which binds to di- and trinucleotides. The EF network confirmed that the ‘*p*-loop’ wave massively recruited loops during a period of over ~ 2.5 Gy of history, especially using pathways of cysteine-rich loop prototypes. In these strong recruitment pathways, the most ancient domains such as NAD(P)-binding Rossmann-fold domain (c.2.1.2) family and the S-adenosyl-L-methionine-dependent methyltransferase domain (c.66.1.43) family, both of which harbored 3-layered α/β/α structures, and the ancient OB-fold of the nucleic acid-binding protein domains (b.40.4.5 and b.40.4.4) with their closed or partly-opened β-barrel structure, enabled many metabolic and translation functions. In particular, the cysteine-rich metal binding loop of the secondarily connected, downstream DS.HE.3.1.1 prototype formed a Zn^2+^-metal binding cysteine nest, which enables interactions with nucleic acids in 6 loop-related domains. This wave also included the class II aminoacyl-tRNA synthetases and biotin synthetases (d.104.1.1) and nucleotidyltransferase (d.218.1.5) families with α/β/α-layered and sheet structures, and beta and beta-prime subunits of DNA dependent RNA polymerase (e.29.1.1 and e.29.1.2) and prokaryotic type I DNA topoisomerase (e.10.1.1) families with β-barrel and winged helix-like structures (Fig. 4A). The terminal DS.HE.2.1 prototype of the cysteine-rich loop recruitment pathway completed the tRNA-independent cysteine biosynthetic pathway 3–3.2 Gya by providing functions to the tryptophan synthase β-subunit-like PLP-dependent domain (c.79.1.1) of serine acetyl-transferase and *O*-acetylserine sulfhydrylase enzymes, reinstating evolutionary analysis of domain organization^64^. The rise of these novelties probably enhanced cysteine availability for binding of Fe-S clusters and recruitment of cysteine-rich loops. These novelties perhaps coincide with the appearance of the PLP-dependent transferase c.67.1.1 domain 3.5 Gy-ago. Finally, the DS.BN.5.5.1 prototype hub also linked downstream glycine and glutamate-rich DS.BK.4.63.1 and DS.BN.6.15.1 prototypes and the upstream glycine-rich nucleotide-phosphate binding DS.HH.1.1.1 that is typically embedded in β/α-barrel structures widespread in metabolism via the Rossmann-like tyrosine-dependent oxidoreductases (c.2.1.2) family structure. Loop DS.HH.1.1.1 was also linked to other downstream prototypes, including DS.EH.4.2.1 and DS.HH.1.1.3 (Fig. 4B).

The second wave in turn originated in the ‘winged-helix’ DNA-binding domain (a.4.5) superfamily (Figs. 4A, 7A), which resurfaced throughout the timeline, starting with the MarR-like transcriptional regulators (a.4.5.28) family ~ 3.3 Gya and its contemporaneous loop hub, the DS.HH.3.1.1 prototype (Figs. 4B, 7B). The wave appeared soon after the *p*-loop wave but part of it merged with the *p*-loop wave through the sister loop hub, the DS.GH.0.0.1 prototype, and its ancestral hub domain, the N-acetyl transferase (d.108.1.1) family. The a.4.5 superfamily harbors the DNA/RNA-binding 3-helical bundle fold (a.4) structure, which is flanked by a 4-strand β-sheet. This domain exposes crucial elbow structures between the helix-turn-helix (HTH) motifs, harboring the specificity of protein–protein and protein-RNA interactions typical of these enzymes. The winged-helix domain is central to transcription^67^. The domain provides nucleic acid clamping and flexibility to RNA polymerases and paired structural recognition interfaces of ubiquitin-ligase and condensing complexes.

Remarkably, these two waves of the EF network and its projections denote the same primordial sandwich α/β/α-layered structures, β-barrels and helical bundle structures referred earlier as part of the first 54 domains that appeared in evolution^37^. The ‘*p*-loop’ and ‘winged-helix’ waves also embedded the first two major gateways of enzymatic recruitment we identified earlier in metabolism^38,66,68^. The first gateway involved the c.37 fold and originated in the energy interconversion pathways of the purine metabolism subnetwork. The second gateway involved the a.4 fold and originated in the subnetwork of porphyrin and chlorophyll metabolism, and the biosynthesis of cofactors. Congruence of this nature obtained using different structural and evolutionary data sets supports our evolutionary statements.

### Modeling the origin and evolution of the ancient domain structures of primordial waves

The earliest polypeptides were likely functionally active prior to the assembly of fully functional protein domains, as recently uncovered by structural relationships of transition metal–ligand binding folds^69^. They would have acted as nucleation foci for construction of larger structures. Phylogenomic data-driven chronologies and networks describe how evolution embeds loops into protein domains. Remarkably, this information allows to model the emergence of folded domain structure by first determining the sequence of events of loop recruitment and then using deep learning algorithms of ab initio structural prediction to find evolutionary patterns of convergence towards the central structural core of the folds.

*P-loop transporters:* To illustrate the power of this approach, we initially focused on the most ancient domain family, the ATP-binding cassette (ABC) transporter ATPase domain-like (c.37.1.12), which is part of the P-loop containing nucleoside triphosphate hydrolases fold (c.37) and superfamily (c.37.1) of SCOP. The fold has a 3-layered α/β/α sandwich arrangement with parallel or mixed β-sheets of variable sizes and topologies. The P-loop containing ABC transporter family that is responsible for the transport of a wide range of molecules across membranes (from small compounds to polypeptides) has a central core with a RecA topology that is missing some typical secondary structures of the fold. Modeling the birth of the fold demanded three procedural steps. First, the times of origin of loops were traced onto the 3-dimensional structure of a representative ABC transporter molecule, as we have previously done with proteins^37^, protein complexes^70^ or the ribosome^71^. Second, a time-ordered series of growing molecules was reconstructed by stitching loop sequences together, starting with the most primordial loop (the P-loop) and adding loops sequentially according to their time of origin. Finally, the three-dimensional structures of the growing molecules were modeled directly from their sequences with AlphaFold2^72^, the star of the last biannual structure prediction experiment (CASP, round XIV)^73^. AlphaFold2 uses a deep leaning algorithm to predict 3-dimensional structure directly from its sequence with levels of accuracy that are within the margin of error of experimental structure determination methods. Calculation of the median ‘global distance test’ (GDT), which measures the similarity of predicted and experimentally acquired structures with known amino acid correspondences, resulted in total scores of well above 90%, indicating global folds and structural details were correct. AlphaFold2 extracts co-evolutionary information in both multiple sequence alignments and structural templates from libraries using an ‘oracle’ that can quickly and iteratively identify which alignment and ‘pair representation’ of structural template data is more informative. This neural network-generated information is then processed by an ‘Evo former’ module to produce increasingly refined deep learning models of both sequence and structure, which converge into the structural prediction. The module uses two attention matrix-based ‘transformer’ architectures to convert the discrete vocabulary of sequence alignments into a continuous ‘embedded’ space of structure capable of training the multiple-layered neural networks. The structural prediction is finally assembled by a ‘structure’ module, which considers a protein as a ‘residue gas’. Each amino acid is modeled as a floating triangle with the three atoms of the backbone, which coalesce into the structure by translations and rotations in space using another attention transformer mechanism and ulterior refinements.

**Figure 8:**
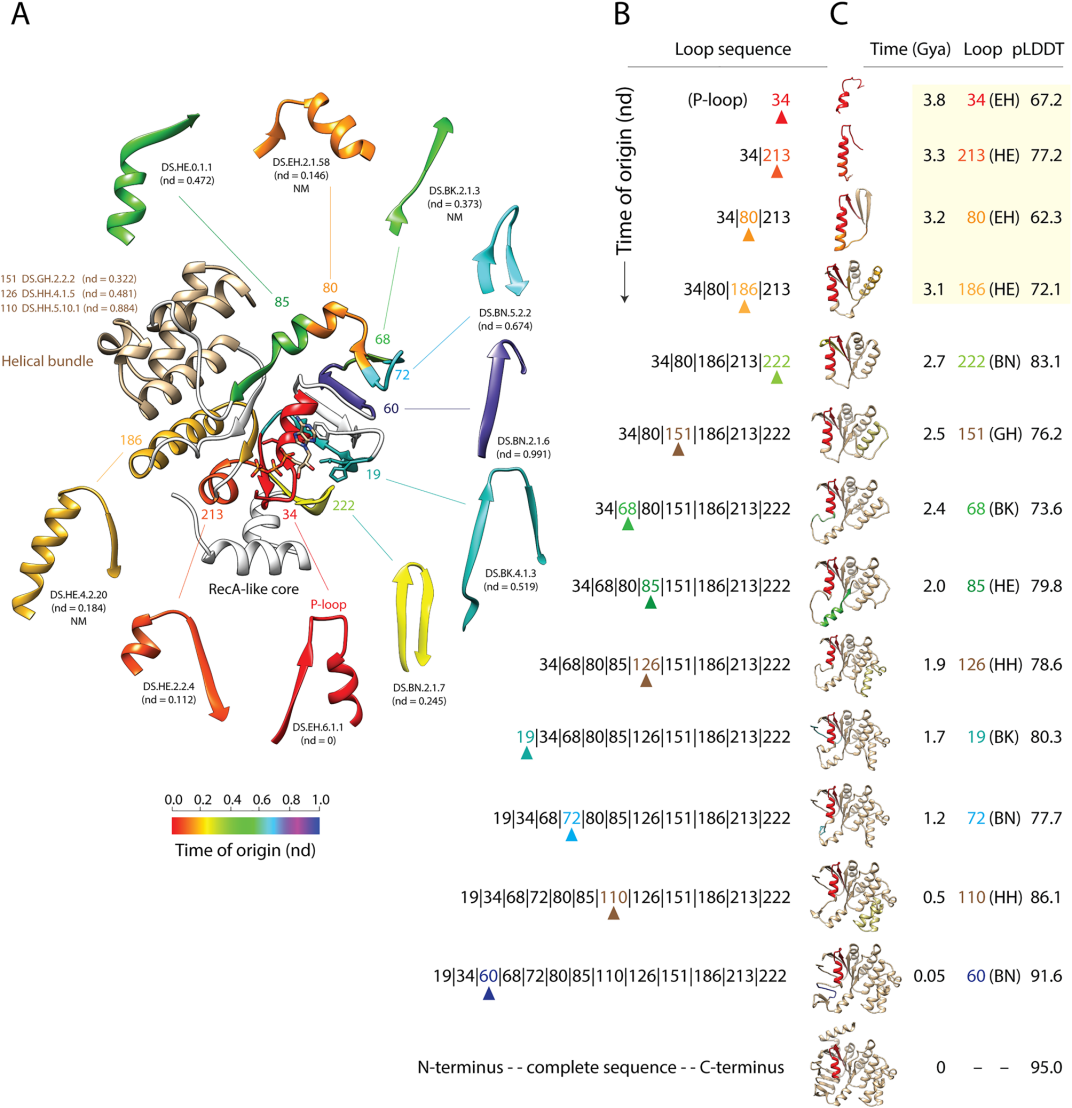
Tracing the evolutionary history of loop prototypes embedded in the structure of the most ancient structural domain. (**A**) A crystallographic model describing the atomic structure of the P-loop containing ATP-binding domain of the histidine permease from *Salmonella typhimurium* (PDB entry 1B0U) shows a nucleotide-binding RecA-like core and an helical bundle region with a signature Mg-ATP binding site that confers substrate specificity. The different loop prototypes that make up the RecA-like core domain structure are colored according to their time of origin, which is given as relative age (nd) in a scale from 0 (origin of proteins) to 1 (the present). They are labeled according to ArchDB classification and mapped to loop structures labeled with numbers describing their position in the sequence relative to the N-terminal amino acid. For simplicity, loop structure numbers and prototypes of the helical bundle are not traced but are listed. (**B**) A time-ordered series of growing molecules was constructed by stitching loops together according to their time of origin. The sequence of loops is given from N- to C-terminus, with loops labeled in numbers and stitching interfaces indicated by pipe symbols. The last loop to be added to the sequence is indicated with an arrowhead and colored according to its age in each model. (**C**) Atomic structures of the growing molecules were modeled directly from their sequences with AlphaFold2. The age of the first loop (the P-loop) and the last loop to be added to the structure are colored in the growing structures. The time of origin (Gya), number label and makeup of bracing secondary structures (in parenthesis) of the newly added loop, and pLDDT confidence level of the ab initio prediction are given for each growing molecule. The time-ordered series of growing structures is shown with larger atomic models in Supplementary Figure S12.

Figure 8 illustrates the results of the three-step strategy. The loop prototypes of the P-loop ATP-binding domain were traced onto the crystallographic atomic structure of a histidine permease enzyme by coloring loop substructures according to the times of origin of their corresponding prototypes (Fig. 8A). These tracings represent a model of accretion of loop substructures in the permease molecule, which in itself becomes a model of structural evolution. The timeline of accretion began with the oldest loop of the molecule, the P-loop (loop structure 34), which mapped to the DS.EH.6.1.1 prototype. The evolutionary growth of the protein was represented as a series of insertions of loop structures in the form of a series of loops [34 > 213 > 80 > 186 > ….] or their corresponding prototypes [DS.EH.6.1.1 > DS.HE.2.2.4 > DS.EH.2.1.58 > DS.HE.4.2.20 > ….]. Alternatively, molecular growth was more appropriately described as a series of molecular intermediates [34 (nd = 0), 34|213 (nd = 0.112), 23|80|213 (nd = 0.146), 24|80|186|213 (nd = 0.184), …], with loop adjacencies in sequences represented with pipe symbols and age (nd) of intermediates given in parentheses (Fig. 8B). This loop sequence representation of growing molecules allows to both track locations of loop insertions at every time step and translate a loop sequence into a series of sequences for AlphaFold2 input. Finally, the ab initio structural predictions produced a series of high-resolution structural representations of the growing molecules, which were placed within a geological time scale framework (Fig. 8C). The per-residue confidence estimate of AlphaFold2 predictions, ranged 62.3–95.0, with values increasing with protein length. This shows confident to very high confident predictions. Since pLDDT assesses local structural accuracy or disorder, Supplementary Fig. S8 shows confidence variation along the protein chain (useful for identifying highly flexible or disordered regions), structural alignments of the 5 ranked predictions produced by the software, and predicted alignment error (PAE) plots measuring confidence in the relative positions of pairs of residues, which is important when evaluating domain packing and large-scale topology.

Remarkably, the series of predicted structural intermediates converged towards a mixed β-sheet flanked by α-helices making up the ‘binding’ cassette of the primordial RecA-like domain core (shaded region of the timeline, Fig. 8C). This occurred within a period of 700 million years spanning 3.8–3.1 Gya (nd = 0–0.184). A three-stranded antiparallel β-sheet already appeared 3.2 Gya in the 3-loop intermediate (23|80|213) hosting the DS.EH.6.1.1, DS.HE.2.2.4 and DS.EH.2.1.58 prototypes, but its structure was likely fluid given analysis of residue pairs in PAE plots and pLDDT variation along the chain (Supplementary Fig. S8). The lone α-helix of the structure belonged to the bracing secondary structures of the P-loop. Further addition of the DS.HE.4.2.20 prototype 3.1. Gya (nd-0.184) rearranged the molecule by adding two α-helices to produce a sandwich structure but converting the initial antiparallel arrangement into a stable parallel β-sheet topology. As accretion proceeded, the fold continued to be accessorized with β-strands and α-helices, which solidified the typical 3-layered α/β/α fold. An extra terminal β-strand in antiparallel arrangement was added 2.7 Gya (nd = 0.245) by incorporation of the DS.BN.2.1.7 prototype, while the previously incorporated DS.HE.2.2.4 prototype gained its C-terminal β-strand ultimately producing a 5-strand mixed β-sheet. PAE plots showed that the 23|80|186|213|222 molecular intermediate appearing 2.7 Gya exhibited a cohesive domain structure. The extra 213 loop structure eliminated the two error-prone bands present in the PAE plots of the prior molecular intermediate (Supplementary Fig. S8). In a next step, an additional β-strand and α-helix were added to the molecule 2.5 Gya (nd = 0.322) following the integration of the first DS.GH.2.2.2 prototype of the helical bundle. This crucial step completed the 6-strand central β-sheet of the extant molecule.

During the initial structural convergence process, there was a temporal sequence of bracing structures of the loops that obeyed molecular elongations matching secondary structures already in place. The sequence followed EH > HE > EH > HE > BN, only stopping by the evolutionary appearance of the first helical component of the bundle 2.5 Gya (nd = 0.322). This steady pattern of ‘reformation’ follows a cryptic phenomenon illustrated by the appearance of the loop structure 213 (mapping to DS.HE.2.2.4) as a helix-coil region 3.3 Gya (nd = 0.122). This integrated structure was reformed into a fluid beta-hairpin 3.2 Gya (nd = 0.146) when pushed towards the C-terminus by the insertion of loop 80 (DS.EH.2.1.58). Its integration onto the expanding β-sheet however was only stabilized into its final form HE, 3.1 Gya (nd = 0.184), once the insertion of loop 186 (DS.HE.4.2.20) reformed the terminal loop 213 structure placing the terminal β-strand at the C-terminal region of the β-sheet. A similar phenomenon occurred 2.4 Gya (nd = 0.373) following the integration of the first α-helix of the bundle. The incorporation of loop 68 (which mapped to DS.BK.2.1.3) downstream the P-loop structure resulted in the formation of a loop form HE, mimicking the DS.HE.0.1.1 prototype (colored green) that was integrated 400 million years later (2 Gya; nd = 0.472). This instance of loop reformation from a BK to HE bracing architecture seems to represent an instance of significant structural rearrangement. These types of rearrangement continue throughout the timeline but are particularly striking during the last 50 million years of evolution (nd = 0.991–1.000) when an entire 4-strand β-sheet was formed. Structural alignments of predicted structures against the extant crystallographic entry showed RMSD values increased from 5.367 to 11.658 Å during the initial convergence period, decreasing thereafter to ~ 4–6 Å and then to 0.75 Å at nd = 1.0 (Supplementary Figs. S8C and Fig. S9). Thus, the central fold design generated during the initial convergence aligns poorly to the modern core, but its folded structure is then significantly optimized during the next 3 billion years of evolution.

Convergence towards the formation of the ‘binding’ cassette of the primordial ABC transporter required a P-loop-centered nucleation of only three loop structures (213, 80 and 186), which are relatively far away from each other in the extant sequence and structure. To test if convergence was resilient, we conducted reshuffling experiments based on the 34|80|186|213 loop sequence where we systematically replaced loop 80 in the second position by all possible loops (60, 68, 72, 85, 110, 126 and 151) that would maintain sequence order, and separately loop 213 in the fourth position by the only option, loop 222 (note that loop 186 could not be replaced). We then modeled structures from the reshuffled sequences and compared them to the reference structure using pruned and total RMSD measurements of structural overlaps (Supplementary Fig. S9). In all cases, reshuffling increased RMSD values significantly, despite changing only one loop in the set of 4 in the experiment (Table 1). Note that reshuffling with loops 68, 72 and 151 (with ages 1.2–2.5 Gya) destroyed completely the β-sheet configuration, while 60, 85, 110 and 126 (with ages 0.05–2.0) preserved it. Reshuffling of two loops in the set of 4 destroyed completely the core structure. These experiments show that the phylogenomic-informed temporal sequence 34|80|186|213 is very sensitive to loop composition, falsifying the notion that the convergence phenomenon towards a fold can occur at random or is an artifact.

**Table 1:** Effect of loop replacement and reshuffling in the structural modeling of the 34|80|186|213 loop sequence.

Loop sequence	RMSD (total atom pairs)
One replacement	
34|**60**|186|213	3.765 Å (73)
34|**68**|186|213	12.441 Å (74)
34|**72**|186|213	14.543 Å (78)
34|**85**|186|213	3.533 Å (77)
34|**110**|186|213	3.342 Å (79)
34|**126**|186|213	3.849 Å (82)
34|**151**|186|213	14.923 Å (80)
34|80|186|**222**	1.569 Å (73)
Two replacements with reshuffling	
34|**68**|**72**|80	16.002 Å (34)
34|**72**|**85**|186	16.664 Å (58)
34|**60**|213|**222**	16.150 Å (52)

RMSD values were calculated for pairwise structural alignments of structural models with modified loop sequences against the reference 34|80|186|213 loop sequence and number of aligned atom pairs described in parentheses. Loops that are replaced or reshuffled are shown in bold in the loop sequence.

*Winged-helix domains:* Winged-helix nucleic acid-binding proteins share a winged helix-turn-helix (wHTH) binding motif made of a right-handed three helical bundle (HTH) and a small β-sheet holding the ‘wings’^67^. The typical three α-helices (α) and three β-strands (β) of the wHTH motif follow the canonical order α1-β1-α2-α3-β2-β3 in the polypeptide chain. They are often preceded by an N-terminal α0 helical extension. While helices α2 and α3 are arranged perpendicular to one another, the nucleic acid-recognition helix α3 makes sequence-specific contacts with the major groove of DNA or RNA. The helix forms hydrogen-bond and van der Waals contacts with functional groups on the exposed base pairs and phosphate backbone. Helices α2 and α3 brace the aperiodic loop region of the very ancient DS.HH.3.1.1 prototype, which defines the nucleic acid-binding specificity of the HTH domain. The two loops that hold the ‘wings’ and a flanking β-hairpin are delimited by β2 and β3 and make other nucleic acid contacts, often with the minor groove or the backbone. Protein–nucleic acid interactions are further stabilized by nonspecific contacts between the nucleic acid backbone and residues in α2 and the turn between α2 and α3. The β-hairpin can separate nucleic acid strands in unconventional helicases holding the wHTH domain^74^.

**Figure 9:**
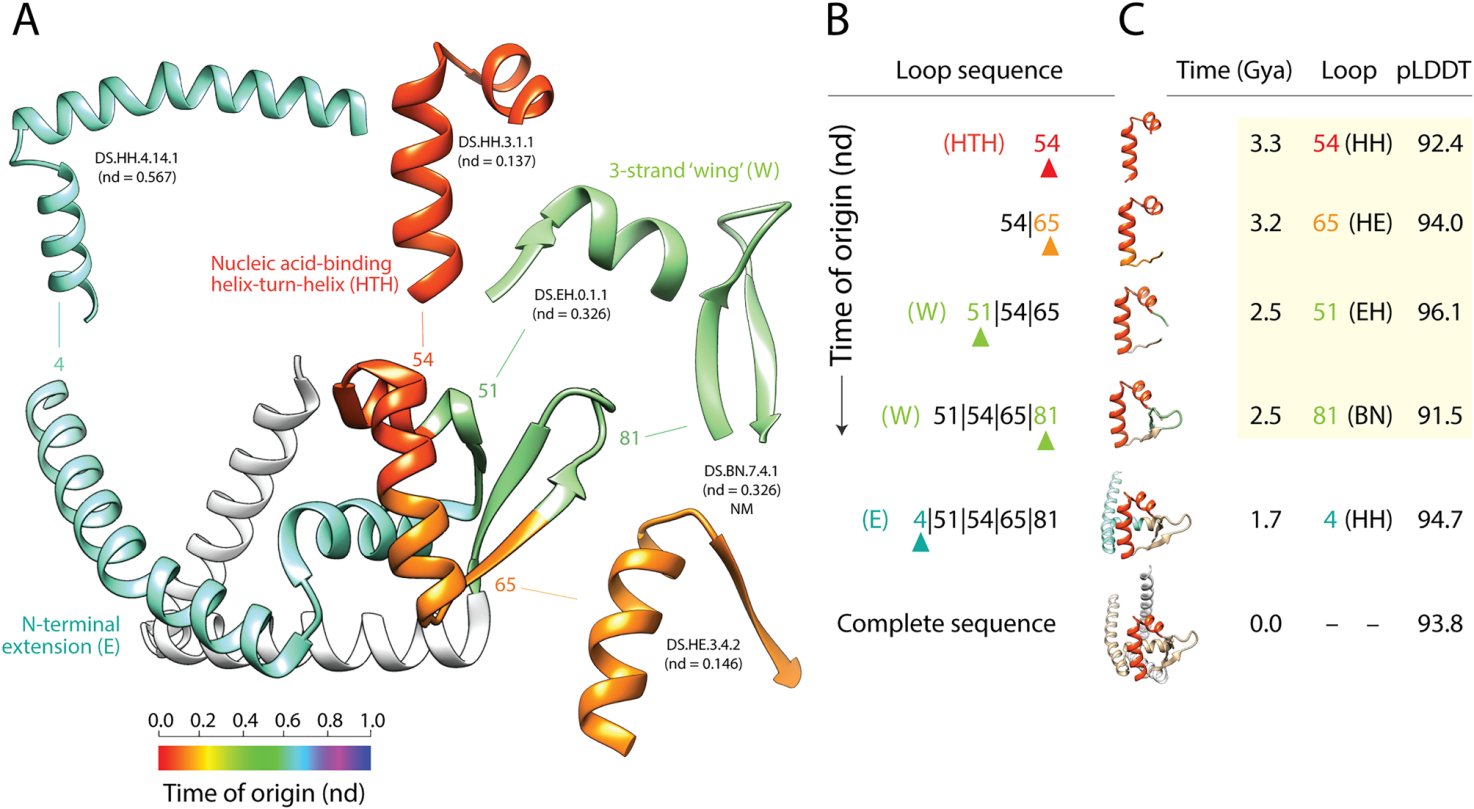
Tracing the evolutionary history of loop prototypes embedded in the structure of the primordial winged-helix domain. (**A**) A crystallographic model describing the atomic structure of the helix-turn-helix (HTH) containing nucleic acid-binding domain of the MarR-type transcriptional regulator mdtR of *Bacillus subtilis* (PDB entry 1S3J) shows the nucleic acid-binding helix-turn-helix (HTH)-containing bundle packed against the 3-stranded β-sheet with ‘wings’ (W) and linked to an N-terminal extension. The different loop prototypes that make up the winged-helix domain structure are colored according to their time of origin, which is given as relative age (nd) in a scale from 0 (origin of proteins) to 1 (the present). (**B**) A time-ordered series of growing molecules was constructed by stitching loops together according to their time of origin. The sequence of loops is given from N- to C-terminus, with loops labeled in numbers and stitching interfaces indicated by pipe symbols. The last loop to be added to the sequence is indicated with an arrowhead and colored according to its age in each model. (**C**) Atomic structures of the growing molecules were modeled directly from their sequences with AlphaFold2. The age of the first loop (the HTH motif) and the last loop to be added to the structure are colored in the growing structures. The time of origin (Gya), number label and makeup of bracing secondary structures (in parenthesis) of the newly added loop, and pLDDT confidence level of the ab initio prediction are given for each growing molecule. The time-ordered series of growing structures is shown with larger atomic models in Supplementary Figure S13.

We modeled the birth of the wHTH fold structure by tracing times of origin of loop prototypes onto the crystallographic atomic structure of the MarR-type transcriptional regulator domain (a.4.5.28) of mdtR of *Bacillus subtilis* (Fig. 9). The timeline of accretion began with the oldest loop of the molecule, the HTH motif (loop 54) that maps to the DS.HH.3.1.1 prototype, and then proceeded with the expected progression of completing the bundle structure of the HTH core, adding the 3-strand wing, and finally the N-terminal extension of the molecule. Again, modeling showed convergence towards the formation of the winged-helix fold, which materialized within a period of 0.8 million years (Fig. 9 and Supplementary Fig. S10). This convergence was nucleated around the ancient HTH nucleic acid-binding loop. Modeling the birth of a wHTH domain variant, a MarR complex of *Staphylococcus aureus*, again revealed an origin in the HTH loop (Supplementary Fig. S11). However, the loop prototypes used to complete the bundle and make up the 3-strand wing were different. The variant added the N-terminal extension first and only started to build the wing ~ 1 Gya, but not fully until 100 million years ago. The two wHTH examples show two convergent evolutionary processes with a single origin (the HTH loop) produced a same fold design. This highlights the central role of recruitment and illustrates how protein folds are permanently revisited by evolutionary convergence.

## Conclusions

The library of single domain protein structures is essentially complete^75^, and so is the library of loop prototypes, which has all geometries sampled^76^, and is considered saturated even for the case of long loops^77^. These properties not only guarantee an exhaustive phylogenomic exploration of the history of domains and loops but also enable the sequence-to-structure mapping of deep learning methods needed to solve the fold recognition problem in ab initio explorations. Here, we trace the history of growing EF networks and their projections, verifying the existence of two primordial waves of functional innovation in elementary functional loops involving founder ‘*p*-loop’ and ‘winged-helix’ domain structures^14^. These waves originally explained recruitment patterns responsible for the origin of modern metabolism^66^. Our findings support an ongoing and highly modular recruitment of loop prototypes into structural domains. Remarkably, we also reveal an underground recruitment process of non-modular loop structures that are drawn at each time event of the timeline. The origin and evolution of loops and domains appears to have evolved in concert from the beginning of the protein world. Structures unfolded at different rates from diverse families of sequence motifs and in different structural contexts. This falsifies the sequential build-up of loops and domains and molecularly ‘canalized’ immutable structures in favor of a dynamic combinatorial landscape of structural creation. Our exploration also supports the evolving EF network becoming structured in evolution by exhibiting hierarchy, modularity, and a power law-based underlying scale free behavior. Integrated communities of interacting structural parts of domains defined modular classes of molecular functions in biphasic patterns of emergence. Collectively, links encapsulated the growth of an elementary alphabet of loop functions embedded in an alphabet of domain structures.

To explore the interplay of these two molecular languages, we modeled the evolutionary emergence of folded structure using historical information derived from chronologies and networks and deep learning algorithms of ab initio structural prediction. We first focused on the oldest domain of the timeline, the P-loop domain of ABC transporters. Remarkably, as accretion proceeded in evolution, the fold converged relatively quickly towards its typical core structure. Convergence, which was organized around the nucleotide-binding P-loop prototype, first materialized into a 3-strand β-sheet, then into the 3-layered α/β/α structure, and finally into an extended central β-sheet forming first a 5-strand and then a 6-strand planar structure. Remarkably, a recent phylogenetic-inspired engineering exploration was able to generate small P-loop–containing loop proteins capable of binding a range of phosphate-containing ligands, including RNA and single stranded DNA^78^. The P-loop prototypes were embedded in a fold made of four tandem β-α repeats with a 3-layered α/β/α sandwich architecture, which much resembled the structural intermediates that appeared 2.7–3.1 Gya in our evolutionary timeline. The study confirmed that short (55-residue) P-loop proteins were catalytically active, supporting the functionality of our early molecular intermediates. Furthermore, construction of 40-residue polypeptides comprising just one P-loop element revealed they acted as helicases capable of separating and exchanging nucleic acid strands^79^ supporting the early nucleic acid-linked functionality of the P-loop prototype. A further focus on the emergence of the winged-helix domain fold of the second wave also revealed quick convergence towards the three helical bundle and 3-strand β-sheet structures of the fold, which centered around the nucleic acid-binding loop. Remarkably, convergence towards the core structure of the winged-helix fold resembled that of the P-loop transporters. In both cases, a central helix component of a functional loop prototype was packed against a small β-sheet structure to enhance functional roles.

Structural convergence suggests the presence of a primordial folding vocabulary in loop structures that overrides the stochastic effects of recruitment. This vocabulary is likely driven by structural reformations occurring within a combinatorial (syntactic) landscape of innovation. Our analysis prompts generalizing ab initio structural prediction of molecular intermediates to all domains in an exploration of how semantics (the meaning of functions and structures) determine the pragmatics (context-dependent rules) of molecular communication.

## Materials and methods

### Phylogenomic analysis and time of origin assignments

Times of origin (age) of domains, were directly derived from a published phylogenomic tree describing the evolution of structural domains defined at FF level of SCOP classification. A calibrated molecular clock of domain structures^36^ allowed calculation of geological ages of FFs in Gy. Details of the phylogenomic reconstruction are provided in Supplementary Materials and Methods. Since a loop is embedded in a domain structure and both loops and domains describe functional and structural abstractions, the age of domains can be directly transferred to loop prototypes. Whenever an older domain donates its loop to a younger domain in evolutionary recruitment, the loop can neither be younger than the younger domain nor be older than the older domain. Consequently, we considered two likely schemes of age transfer: (1) the age of a loop is the age of the most ancient associated domain, or (2) the age of a loop is the age of the more recent of the pair of most ancient associated domains. The age of a loop prototype in these schemes is either the age of the first structural scaffold or the age when the loop function is first transferred between structural scaffolds, respectively. Both schemes provided similar age mappings. For that reason, we only present mappings derived using the second more conservative scheme. Since the first loop that appeared in evolution must generate the first domain in order to preserve the *‘lex continui’* principle and a donor loop has to be either older or at least contemporaneous to the acceptor domain, the most ancient loop DS.EH.6.1.1 was assigned a time of origin of 0 according to scheme (1) as an exception because of the absence of any older donor domain.

### Domain and loop prototype data

Loop prototypes were computationally identified by filtering DS-derived loop structures from ArchDB^27^ while mapping domains to loops at e-value < 0.001. This resulted in 88,321 loops structures clustering into 7078 unique loop prototypes that mapped to 2447 domains, with 9650 mappings. Note that each loop structure in ArchDB has one loop prototype annotation in the DS classification system with many-to-many mappings between loops and domains. Out of the set of 7078 loop prototypes, a subset of 5125 only mapped ‘horizontally’ and uniquely to domains of a same age within a subset of 1965 domains. They were reported as *‘non-modular’* (NM) loop prototypes because they failed to be recruited by different domains across the timeline, including those that were contemporaneous. In contrast, a subset of 1937 loop prototypes mapped ‘vertically’ to 1442 domains with times of origin spread throughout the timeline. They were reported as *‘modular’* (M) loops since they acted as modular units of structural, functional, and evolutionary significance. Finally, a subset of 16 loops each mapped ‘horizontally’ to sets of 2 contemporaneous domains, a subset of size 32. We reported these loops as *‘modular’* (contemporaneous) (M’) loops because they involved recruitments occurring within individual time events and representing focal innovations.

### Network visualization and analysis

Networks were visualized and analyzed using Pajek^80^ and R’s *igraph* package^81^. Community-based layouts of the networks were generated using the Visualization of Similarity (VOS) clustering method. Network properties were analyzed with code constructed using graphing packages and tools of R^82,83^. A detailed description of data files, partitions and functions used to analyze network data, produce charts and plots, compute power law statistics and modularity indices, and construct waterfall diagrams can be found in the Supplementary Materials and Methods.

### Statistical analysis

#### Power law network behavior

Scale free network behavior was studied using *P(k)* vs. *k* (probability of having *k-*neighbors vs. *k*) and *log–log* (log of *P(k)* vs. log of *k*) mathematical curves, with linear regression models to derive γ of the power law and the determination coefficient (R^2^). γ is the absolute slope of the log linear model. Higher γ indicate higher levels of preferential attachment. R^2^ describes the percentage of the data fitting the linear model. High values of both γ and R^2^ indicate that scale free behavior is strongly supported. Other power law statistics included: (1) KS fit statistic, which compares the fitted distribution with the input degree vector; (2) the KS *p*-value, with the null hypothesis of data being drawn from the power law distribution^62,63^; and (3) the exponent of the fitted power law distribution (α), which assumes P(*X* = *x*) is proportional to x^–α^. Lower KS fit score, larger KS *p*-value (≥ 0.05), and higher α suggest better fit to power law distribution. The maximum log likelihoods of the fitted parameters were also determined. Reference networks were created using ‘Barabási’ methods^84^ of R’s *igraph* package^81^ to simulate power law and extended age-dependent control models for the corresponding networks.

#### Network modularity

We studied modularity with six indices: (1) The *VOS Quality index* (*VQ*), was generated by the Pajek layout algorithm that considers weights of links (edges/arcs) as similarities. Communities were iteratively drawn closer based on similarity and the quality index of the final layout with least crossings and closest clusters was given. *VQ* is then calculated as ∑_i=1àc, j=i+1àc_ (e_ij_ – a_i_^2^), where c is the number of communities. e_ij_ is the fraction of edges with one node v in community i and the other w in community j, given as ∑_vw_ (A_vw_/2 m) with 1_v ϵ ci_, 1_w ϵ cj_, where m is the sum of weights in the graph and A_vw_ = the weighted value or 0, indicating presence or absence of edge between the nodes v and w, respectively, in the adjacency matrix A of the network. Finally, a_i_ is the fraction of weighted k neighbors that are attached to the nodes of a community i, i.e. k_i_/2 m ^46,47^; (2) The *Clustering Ratio* (*C-ratio*) is the ratio of the number of clusters to the size of an inter connected node set; (3) The average *Clustering Coefficient* (*C*) describes the mean ratio of triangles to connected triads over all nodes in the simplified (undirected/unweighted) network^52–54^ is meaningful only for unimodal graphs^62^. We also report coefficients of linear regression over *C* for loop and domain network projections; (4) The *Fast Greedy Community* (*FGC*) hierarchical agglomeration algorithm detects community structure with linear run time O(m d logn) ~ O(n log^2^n), of a network with m edges, n nodes, and depth d of the dendrogram describing its community structure^63^; and (5 and 6) The *Newman-Girvan* algorithm index (*NG*), computed with partitions defined by age (*NG*_*age*_) and VOS clustering (*NG*_*vos*_). *NG* calculates the modularity of a network based on some classification (partition) to measure how good that classification is in dividing the various node types, indicated by assortative (positive) or disassortative (negative) mixing across modules^51^. *NG* equals 1/(2 m)∑_ij_(A_ij_ − 1/(2 m)k_i_k_j_*∆(c_i_,c_j_)), where m is the collective weights in the graph, A_ij_ are weighted entries in the adjacency matrix, k_i_, k_j_ and c_i_, c_j_ are the weighted degrees and the components (numeric partitions), respectively, of nodes i and j each, and finally, ∆(x,y) is 1 if x = y and 0 otherwise^57^. *VQ*, *C-ratio*, *C* and *FGC* each range from 0 to 1, while the *NG* indices range from − 1 to 1. Higher indices represent strong network modularity at an event. Heatmaps were generated using customized scaled modularity matrices with elements given as (A_ij_ − k_i_k_j_/(2 m))M_*nd*_, where A_ij_, k_i_, k_j_ and m are as defined for *NG*^51^, and M_*nd*_ is a network’s modularity index at event *nd*. Dendrograms were calculated as squared Euclidean distance matrices indicating dissimilarities between the cluster means^85^. The distance (or dissimilarity) matrices were hierarchically clustered with the Ward's minimum variance method aiming at finding compact, spherical clusters^86^.

### Ab initio modeling

The 3-dimensional structures of evolving molecules were modeled directly from their sequences with the AlphaFold2 pipeline^72^ in ColabFold^87^. We requested output of 5 ranked models obtained with 3 recycles using PDB70 as template and the multiple sequence alignment (MSA) mode MMseqs2 (UniRef100 + Environmental). The use of PDB70 template did not significantly affect modeling results. Accuracy was measured with pLDDT and the predicted aligned error (PAE). pLDDT provides a per-residue estimate of prediction confidence based on the local Distance Difference Test (lDDT)-Cα metric^88^. The expected prediction reliability of a given region or molecule follows pLDDT ‘confidence bands’: > 90, models with very high confidence; 90–70, models with confidence, showing good backbone predictions; 70–50; models with low confidence; and < 50, models with very low confidence, generally showing ribbon-like structures. pLDDT < 60 can be considered a reasonably strong predictor of intrinsic disorder. TAE measures confidence in the relative positions of pairs of residues and is a good metric to evaluate the cohesiveness of domains. Structural alignment were carried out using subroutines of the visualization software Chimera^89^ (available at https://www.rbvi.ucsf.edu/chimera). Frag’r’Us was used to sample protein backbone conformations of loops^90^.

### Supplementary Information

Supplementary Information 1.

Supplementary Information 2.

Supplementary Information 3.

Supplementary Video 1.

Supplementary Video 2.

Supplementary Video 3.

Supplementary Video 4.

Supplementary Video 5.

Supplementary Video 6.

Supplementary Video 7.

## Data Availability

The data that supports the findings of this study are publicly available in the ArchDB (http://sbi.imim.es/archdb/), SCOP (https://scop.mrc-lmb.cam.ac.uk) and SCOPe (https://scop.berkeley.edu) repositories. AlphaFold2 modeled structures have been deposited in ModelArchive (https://www.modelarchive.org) under global accession code ma-gca-proto. Other data and information supporting the findings of this study are available within the article and its supplementary information files.
