# Supplementary material for: Tracing the birth of structural domains from loops during protein evolution
Source: Sci Rep. 2023 Sep 6;13:14688. doi: 10.1038/s41598-023-41556-w (PMC10482863; doi:10.1038/s41598-023-41556-w)
Supplement: Supplementary file 1 — Supplementary Information 1. [file 41598_2023_41556_MOESM1_ESM.pdf]

SCIENTIFIC REPORTS

Supplementary Materials for

Tracing the birth of structural domains from loops during protein evolution

M. Fayez Aziz[1], Fizza Mughal[1] and Gustavo Caetano-Anollés[1,2]*

[1]Evolutionary Bioinformatics Laboratory, Department of Crop Sciences, University of Illinois, Urbana, IL 61801, United States; [2]C. R. Woese Institute for Genomic Biology, University of Illinois, Urbana, IL 61801, United States
*Correspondence and requests for materials should be addressed to G.C.A (e-mail: gca@illinois.edu

The PDF file includes:

Text S1. Supplementary materials and methods
Fig. S1. Various network forms, features and layouts illustrated through a model miniature network.
Fig. S2. Statistical descriptors of power law behavior for the EF bipartite network and its loop and domain network projections.
Fig. S3. Modularity indices of the EF bipartite network and its loop and domain network projections.
Fig. S4. Hierarchical organization of the fully-grown (extant) EF network (nd=1.000).
Fig. S5. Boxplots of the EF bipartite network (A) and its loop (B) and domain (C) network projections.
Fig. S6. Connectivity of events along the timeline in the loop (A) and domain (B) network projections of the EF network.
Fig. S7. Measure of central tendency and dispersion in loop and domain components of the EF network.
Fig. S8. Confidence parameters of AlphaPhold2 structural predictions of a P-loop transporter.
Fig. S9. Structural alignment of modelled structures to the crystallographic entry of the P-loop transporter (PDB 1B0U).
Fig. S10. Confidence parameters of AlphaPhold2 structural predictions of the winged-helix transcriptional regulator mdtR.
Fig. S11. Tracing the evolutionary history of loop prototypes embedded in the structure of a variant of the primordial winged-helix domain.
Fig. S12. A time-ordered series of growing structures and corresponding atomic models of the P-loop containing ATP-binding domain.
Fig. S13. A time-ordered series of growing structures and corresponding atomic models of the winged-helix domains of two transcriptional regulator types.

Other Supplementary Material for this manuscript includes the following:

Table S1. Loop-centric data.
Table S2. Domain-centric data.
Video 1. Simulation of pairwise NGage modularity of the EF network.
Video 2. Simulation of hierarchical organization of the EF network.
Video 3. Simulation of waterfall model of the loop network projection.
Video 4. Simulation of waterfall model of the domain network projection.
Video 5. Simulation of network growth in the EF bipartite network.
Video 6. Simulation of network growth in the loop projection network.
Video 7. Simulation of network growth in the domain projection network.

Text S1. Supplementary materials and methods

Phylogenomic analysis. We conducted a census of structural domains defined at FF level of SCOP classification in 8,127 proteomes of completely sequenced genomes from cellular organisms and viruses[29] using profile hidden Markov models of the SUPERFAMILY database with assignments at e <0.0001[89]. Proteomes analyzed were from 139 archaeal, 1,734 bacterial, and 210 eukaryal representative and reference cellular organisms sampling all major taxonomic groups of the RefSeq database[88]. We also included a set of non-redundant 6,044 viral proteomes after exclusion of unclassified viruses, which were categorized according to genome type and viral replication using the Baltimore classification. Phylogenomic analysis was carried out as previously described[90] according to verified phylogenomic protocols[18]. Briefly, the abundances of individual FFs in every proteome were log-transformed and rescaled by dividing by the maximum abundance in that proteome followed by additional rescaling to 24-character states to ensure compatibility with PAUP* (version 4.0)[91]. Normalization and rescaling protected against the effects of unequal proteome sizes and variances. Maximum parsimony (MP) was used to reconstruct optimal phylogenomic trees of domains describing the evolution of FFs (taxa) using proteomes as characters. Trees were rooted using the Lundberg method in PAUP*, which does not require specification of any outgroup taxon and complies with Weston's generality criterion of phylogenetic rooting. Instead, an unrooted network is calculated, which is rooted a posteriori by the branch yielding a minimum increase in tree length. We note that MP approximates maximum likelihood when phylogenetic characters evolve at different rates[92] and is appropriate for global proteome studies. Bootstrap analysis with 1,000 replicates was performed to assess the reliability of deep evolutionary relationships. Trees were visualized with FigTree version 1.4.4 (available at http://tree.bio.ed.ac.uk/software/figtree/). A single tree of domains reconstructing the evolutionary history of 3,892 FFs in the 8,127 proteomes, which was recovered from a heuristic search (Fig. 1C), was used to determine the times of origin of domains by calculating *node distance* (*nd*) values directly from the rooted tree. A calibrated molecular clock of domain structures was used to calculate geological age in Gy[31].

Network data analyses. The EF network and the loop and domain network projections were visualized and analyzed using Pajek[75] as they unfolded in the evolutionary timeline. The nodes (vertices) and links (arcs/edges) of the EF bipartite graph and its projections were encoded in Excel (ASCII) project files for network visualization. The quantitative network property of cumulative weighted degree per node (*cwdn*) was generated using custom Pajek macros. *cwdn* was compiled as three types of data matrices for each network (BOX 1). The data rows of matrices defined loops and domains, and were sorted in ascending order of node-(individual *nd*) or network-(event *nd*) age. Separate matrices were organized for the 'in' and 'out' degree types as well as each portion of the bipartite node set.

BOX 1. Types of data matrices

Matrix type	Matrix objective
By 'node age' (NOA)	Used for box plots and x-y line plots. Categorical columns were ordinal number, age bin, age and node label of loop and domain nodes in the networks. Additional columns described *cwdn* in increasing order of events. Rows were sorted by node.
By 'network age' (NEA)	Used for power law distribution graphs. They are essentially transpositions of NOA data. Columns were ordinal number, age bin, and age of networks, followed by *cwdn* arranged by nodes. Rows were sorted by events.
By 'degree dispersion' (DD)	Used for stacked bar charts. Column and row order are the same as NOA data. However, columns provided the distributions of final *cwdn* (i.e. at *nd*=1) across connected node age bins.

Network visualization. A set of Pajek menu commands was used to generate visualization attributes and layouts (BOX 2). Node categories were made distinguishable using various shape and color options. Evolutionary patterns were unfolded by color-coding the age of loop and domain nodes. A color palette was used that ranged from red for the most ancient node (*nd* = 0) to blue for the most recent node (*nd* = 1).

BOX 2. Network visualization tools and commands

Pajek tool and command	Output
Network: Create Vector: Centrality: Weighted Degree: 'All', 'Output\Input'	The weighted degree vectors for undirected and directed networks, respectively.
Draw: 'Network + Vector'	Visualization of the weighted degree of nodes as node size.
Network: Create Partition: Communities: VOS clustering: 'Multi-level coarsening + Multi-level refinement'	The community-based layout of the networks, using the VOS clustering method[41,42]. In addition, modularity indices were obtained. Default parameters were used.
Draw: 'Network + Partition' and Draw: Layers: 'In y Direction'	Vertical arrangement of nodes according to their age in both bipartite and waterfall layouts of the networks with age mappings.
Draw: Layout: Energy: Kamada-Kawai: 'Optimize inside clusters only'	Visual distribution of partitions[43] (clusters or communities) in waterfall layout, manually separated to refine the most energetically favored network configurations.

Charts and graphs. Graphing code constructs and packages of R[79,80] were used to visualize the *cwdn* (BOX 3).

BOX 3. Graphing and charting constructs and operations

R *package*: function	Result
*Reshape: **	Transform textual data tables to vector form.
*Lattice: **	Generate panels of network- and node-age bins.
*LatticeExtra, grid: **	Resize panels.
LatticeExtra: '`panel.lmline` ()'	Draw linear regression model lines and equations, e.g. in *log-log* graphs.
*Data.table: **	Edit data tables, e.g., to remove empty age bins.
*igraph: **	Read network graph files to calculate modularity and apply the power law distribution model to calculate its fit statistics.
R base: '`bwplot` ()'	Box and whisker's plots based on NOA files, to depict measures of central tendency of *cwdn* over network age.
Lattice: '`xyplot` ()'	XY scatter and line plots based on NOA files drawing final *cwdn* attained at network age 1.
*R color palette: **	The color-coding of data points by node age.
ggplot2: '`ggplot` ()', '`geom_bar` ()' and '`grid.draw` ()'	Produce degree dispersion stacked bar charts and graphical trend curves of power law behavior and modularity, based on DD, NEA data and VOS modularity report files, respectively.
ggplot2: `scale_fill_manual` () `scale_color_manual` ()	Color-coded stacked bars representing distribution of final *cwdn* over ages of connected node set.
ggplot2: '`layer` ()'	Stack negative valued bars in reflection.
ggplot2: '`theme` ()'	Color-coding of multiple curves of power law statistics and modularity indices.

gridExtra: '`grid.arrange` ()' and '`arrangeGrob` ()'	Plotting together of curves for comparison.
ggplot2: '`geom_smooth` ()'	Addition of linear regression lines to some modularity plots.
ggplot2: '`geom_tile` ()', '`scale_fill_gradient` ()', '`scale_x/y_discrete` ()' etc.	Customized modularity heat maps.
*ggdendro: **	Plotting of dendrograms.
R base: '`aggregate` ()'	Calculate averages of categorized data.
plotrix: '`std.error` ()'	Measure dispersion of data through standard error.

Barabási reference networks. Reference networks for comparative analysis of power law and modularity were generated using 'barabasi'[81] methods of the R's *igraph* package[76]. The ideal power law model was implemented with 'barabasi.game ()'. An extended model was implemented with 'aging.prefatt.game ()'. This model simulated the scale-free evolution of random graphs by altering the probability of preference of an old vertex growing multifold, exponentially with age. Networks of three sizes, 3379, 1937 and 1442, were created to simulate reference controls for the corresponding EF, loop and domain networks, with properties of directionality directly transferred. Ages were assigned to individual nodes in incremental order to keep age proportion per event consistent with the timelines of the test networks.

Power law statistics. The gamma coefficient from *log(P(k))* vs. *log(k)* data provided preliminary insight into the scale free behavior of a network. Power law behavior usually manifests in degree distributions exhibiting long tails and a low deviation from power law fit. R libraries and operations were utilized to run the analysis (BOX 4). The distributions were color-coded.

BOX 4. R operations used in power law analysis

R *package*: function	Result
Lattice: '`xyplot` ()'	Plot distributions generated from NEA files.
Data.table: '`table` ()'	Compute *k* frequency tables for *P(k)* vs. *k* plots.
R base: '`length` ()'	Calculate length of degree table in order to determine *P(k)* through quotient of *k* frequencies by maximum degree.
R base: '`log` ()'	Calculate *log P(k)* vs. *log k* data to obtain gamma coefficient.
Lattice: '`lm` ()' and '`panel.abline` ()'	Generate and draw linear regression models.
R base: '`summary (lm)$ coefficients`'	Retrieve the γ-slope power law coefficient and R^2 determination coefficient.
igraph: '`power.law.fit` ()'	Obtain additional statistics supporting the preferential attachment principle.

Modularity indices. Modularity indices were calculated for each network using various capabilities of the *igraph* package[76] (BOX 5). Isolated vertices were deleted using a custom function and corresponding partitions were adjusted, as required by the modularity algorithms. We skipped modularity computation of the empty (no-node or no-arc) network instances until *nd* ~ 0.0043 in loop_domain bipartite network, and until *nd* ~ 0.0086 in loop and domain projection networks to avoid computing errors. *cwdn* was used as input in calculation of all modularity indices. VOS modularity indices and partitions were generated using Pajek[75]. *NG* modularity indices[50] were computed using two types of memberships (or partitions) as input: VOS clustering (NG_{vos}) and age (NG_{age}). Clustering ratios were determined using custom functions

by dividing the number of clusters from the connected node set with the size of the connected node set.

BOX 5. R's *igraph* package operations used in modularity analysis

igraph's function	Result
'read.graph ()'	Import network graph data.
'fastgreedy.community ()'	Calculate the *FGC* modularity index[58].
'as.undirected ()'	Collapse directed edges for *FGC.*
'modularity ()'	Compute *NG* modularity indices.
'transitivity ()' with the 'average' option	Determine average clustering coefficient (C)[48,53,53].

Heatmaps and dendrograms. *NG* pairwise modularity matrix was computed for each network using 'mod.matrix ()'[49]. Three types of memberships (or partitions) were used as input: VOS, age and *FGC*. *FGC* partition was extracted by 'membership ()'. Each matrix was scaled by the absolute log10 value of the overall modularity score of the network, before being drawn as a heatmap. Pairwise scores were saturated to the range [-1, 1]. The x-y axis node labels were color-coded and ordered by age. In dendrograms, the x-y axis node ticks were color-coded by age and labels were color-coded by bipartite node type. Dendrograms were generated through 'dist ()' by calculating squared Euclidean distance matrices that indicate dissimilarities between the cluster means[82]. The distance (or dissimilarity) matrices were hierarchically clustered using 'hclust ()' with the Ward's minimum variance method aiming at finding compact, spherical clusters[83]. Nodes that were clustered in the dendrograms were ordered within clusters by age and were color-coded as such.

External support. Additional information, including scripts and workflows for visualization and statistical analyses can be found in the Molecular Ancestry Networks (MANET) database repository (http://manet.illinois.edu).

Supplementary references

91. Swofford, D.L. 2022. Phylogenomic Analysis Using Parsimony and Other Programs (PAUP*) Ver 4.0b10. Sinauer, Sunderland, Massachussetts.
92. Kolaczkowski, B., Thornton, J.W. 2004. Performance of maximum parsimony and likelihood phylogenetics when evolution is heterogeneous. Nature 431, 980–984.

Supplementary Figures

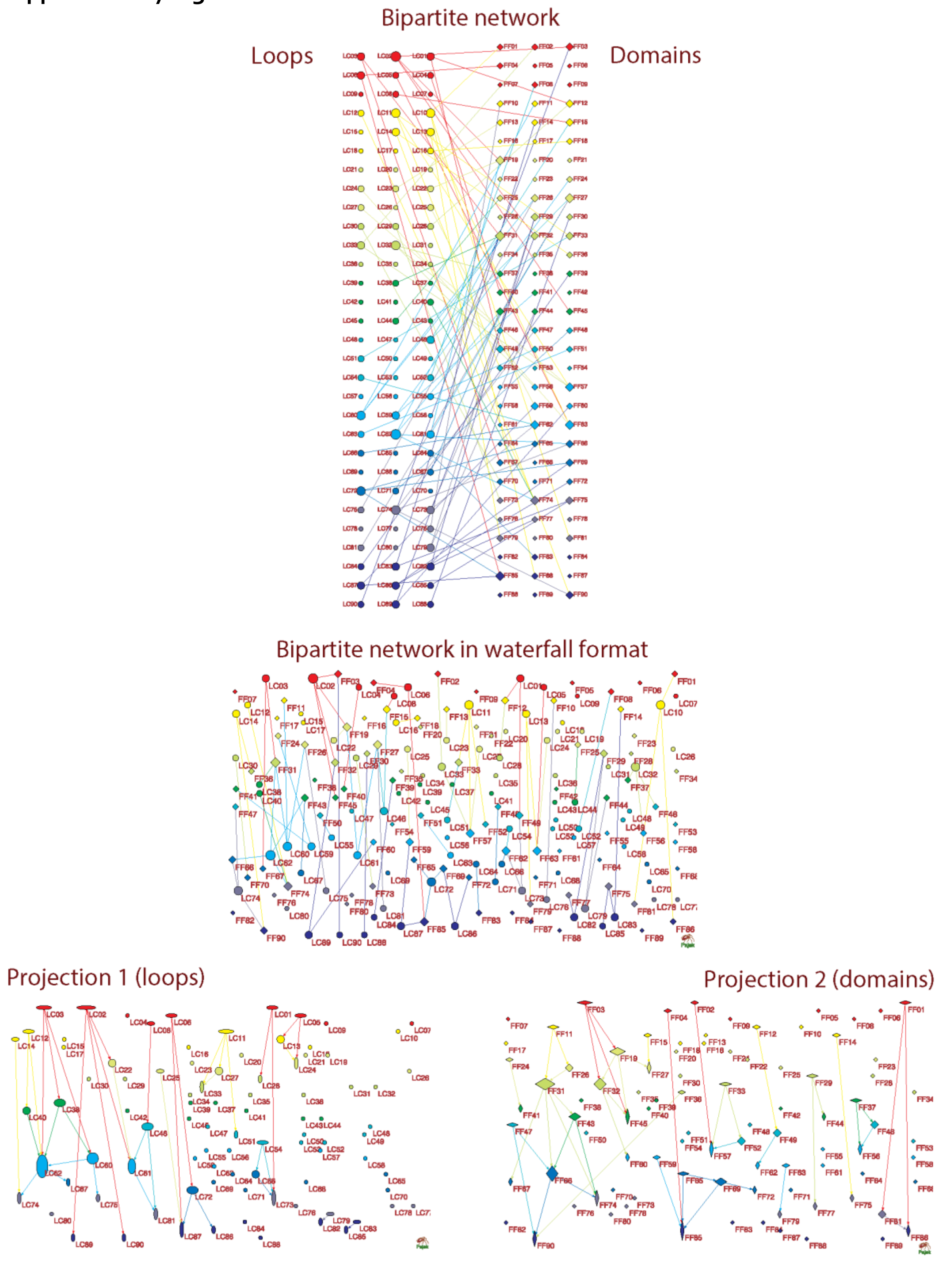

Figure S1. Various network forms, features and layouts illustrated through a model miniature network.

(Figure S1 legend continued from previous page) The diagrams illustrate an undirected bipartite miniature network in bipartite **(A)** and watrefall **(B)** layouts its two network projections in waterfall layouts **(C,D)**. The original form has 90 nodes per bimodal parition and 30 nodes per age, resulting in a reduction in number of corresponding node groups by one-third if shrunk by age. Loop nodes are designated as 'loop classifiers' (LC). Domain nodes are designated 'fold families' (FF). They are described with symbols, with size proportional to the number of links they establish. The connectivity in the undirected bimodal network was generatd at random, strictly between the two paritions. The connectivity in the projections was generated based on the sharing instances within partitions and describes construction of directed 'discrete event' networks in waterfall format. As time progresses from top to bottom in a waterfall layout, events pan out as the progressive appearance of nodes and links, clustered from left to right. Network growth was made explicit by coloring of nodes according to age (red-to-blue) and that of links by node of origin, by using time-induced arrows or arcs (edges in case of undirected) with density proportional to node connectivity, and by maintaining horizontal and vertical sizes of symbols proportional to the respective alldegree, outdegree and indegree of the nodes. As the network grows with time, the transition from wide to tall symbols facilitates the visualization of the source-sink origination dynamics of recruitments in the projections, with chronological accumulation of connections increasing outdegree of an earlier originating node and widening the node-symbol horizontally, while increasing the indegree of a later receiving node and lengthening the vertical scale of the node-symbol.

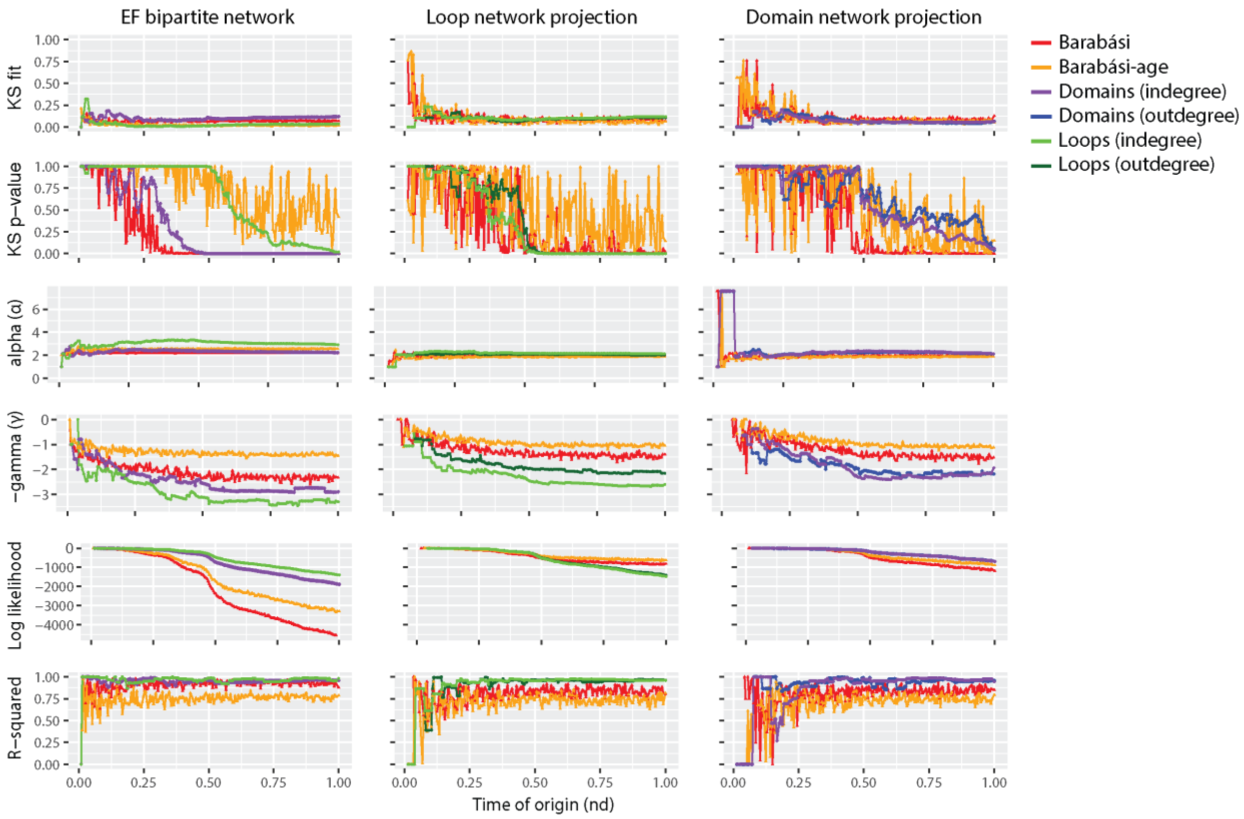

Figure S2. Statistical descriptors of power law behavior for the EF bipartite network and its loop and domain network projections. Six differential indicators of preferential attachment were plotted for each network. Two reference curves, Barabási (red) and Barabási-Age (orange), were included for comparison. Barabási specifies the probability of preference of an old node as $P_i \sim k_i^\alpha$ while Barabási-Age confers heavier power law properties to older (with smaller *nd*) nodes using $P_i \sim (k_i^\alpha)(l_i^\beta)$. Here, k_i is the indegree of node i in the current event; α is the preferential attachment exponent, with default value one, i.e. linear preferential attachment; l_i is the age of node i, i.e. the number of events elapsed since the node was added, with maximum number measured by the 'aging.bin' parameter; β is the exponent of aging, kept at 1 for linear increases in probability of preference of an older node (with high l_i). Separate curves were computed for the 'alldegree' of loop and domain portions of the EF network and for 'outdegree' and 'indegree' of projected loop and domain networks. Degrees were cumulative and weighted. The six power law indices include: (**i**) the KS fit statistic that scores deviation of input degree data vector from the fitted power law distribution; smaller scores denote better fit; (**ii**) the KS p-value less than α=0.05 indicate rejection of the null hypothesis of degree data being drawn from the fitted power-law distribution[46,47]; (**iii**) the exponent of the fitted power-law distribution (α); (**iv**) the slope of power-law linear regression model (-γ); (**v**) the log-likelihood of the fitted parameters (likelihood); and the (**vi**) coefficient of determination (R^2) indicating confidence in the linear model through percentage of degree data explained by the regression line. Higher α, lower -γ and closer to zero likelihood correspond to power law behavior. Statistics were calculated for each event of the growing networks. Time of origin (*nd*) is indicated on a relative 0-to-1 scale.

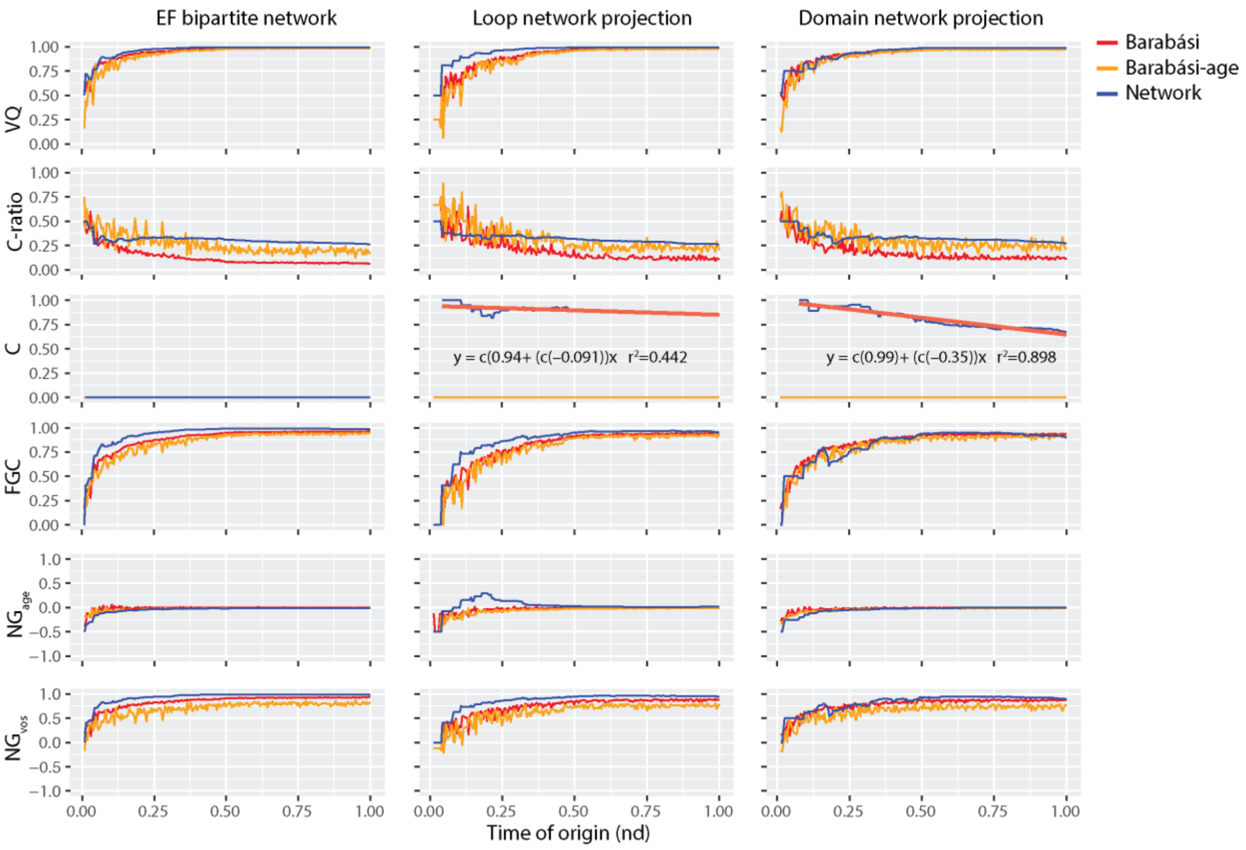

Figure S3. Modularity indices of the EF bipartite network and its loop and domain network projections. Six indicators of modularity were plotted for each network. The coefficients of linear regression lines (grey) over *C* for the loop network were -0.000033 by network size (*N*) and -0.091 by age, and those for the domain network were -0.000190 by *N* and -0.350 by age. Determination coefficients (R^2) were 0.372, 0.442, 0.960 and 0.898, in that order. The linear models shown are by *nd*. Two modeled control sets, Barabási (red) and Barabási-Age (orange) were included as reference. The "Age" control enriches preferential attachment properties in older (smaller *nd*) nodes, inducing slightly different modularity curves. Single comprehensive modularity curves were computed for each network. Modularity calculations required cumulative and weighted connectivity input. Age (*nd*) is indicated on a relative 0-to-1 scale.

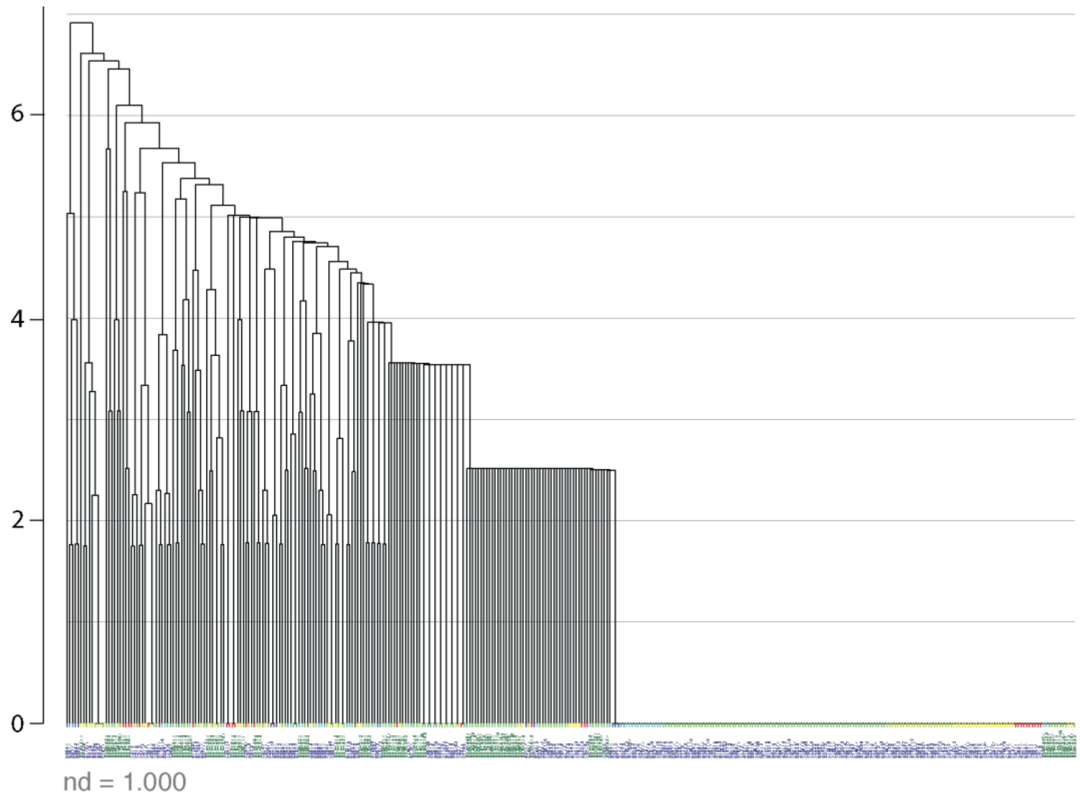

Figure S4. Hierarchical organization of the fully-grown (extant) EF network (*nd*=1.000). NG_{age} scaled modularity matrix[50] of the last distinguished EF network in the timeline was input to calculate Euclidean distance matrix[82] which was hierarchically clustered with the Ward's minimum variance method[83]. Vertical axis represents dissimilarity between clusters of the dendrogram. Horizontal axis labels are color-coded to distinctively identify the rearrangements of the two classes of constructs, loops (green) and domains (blue). The nodes are age-sorted ascendingly within clusters, color-coded by node age and labelled using standard SCOP nomenclature[28]. The most ancient loop motifs and domain structures of the clusters comprising 95% percentile connectivity of the two major waves of functional innovation and the hidden EF switch of power law and modularity exchange are displayed on the tips of clades.

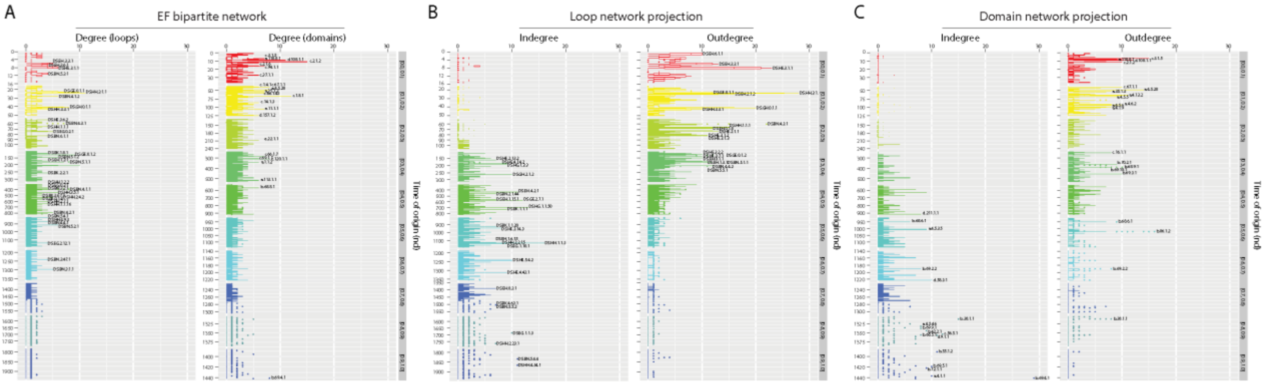

Figure S5. Boxplots of the EF bipartite network (**A**) and its loop (**B**) and domain (**C**) network projections. The Tukey boxplots show boxes (first and third quartiles bracketing the median), whiskers (values within ±1.5 × inner quartile range), and outliers. The plots describe the span of connectivity given as expansion in degree of each node along the events of evolutionary timeline. They provide a view of chronological accumulation of connections (in the form of links or arcs) with time. For the EF network, connectivity of loop and domain portions is given separately. For the projected loop and domain networks, arc connectivity is given as node indegree and outdegree and plotted separately. Each event corresponds to the discovery of loops and domains, labeled using standard SCOP nomenclature[28], from one of 206 and 226 events, respectively, along a timeline labeled using relative 0-to-1 scale on the right of the plots. Significant nodes in the 95th percentile of connectivity were promptly labelled. The timeline of events was coarse-grained into 10 age bins (colored red-to-blue).

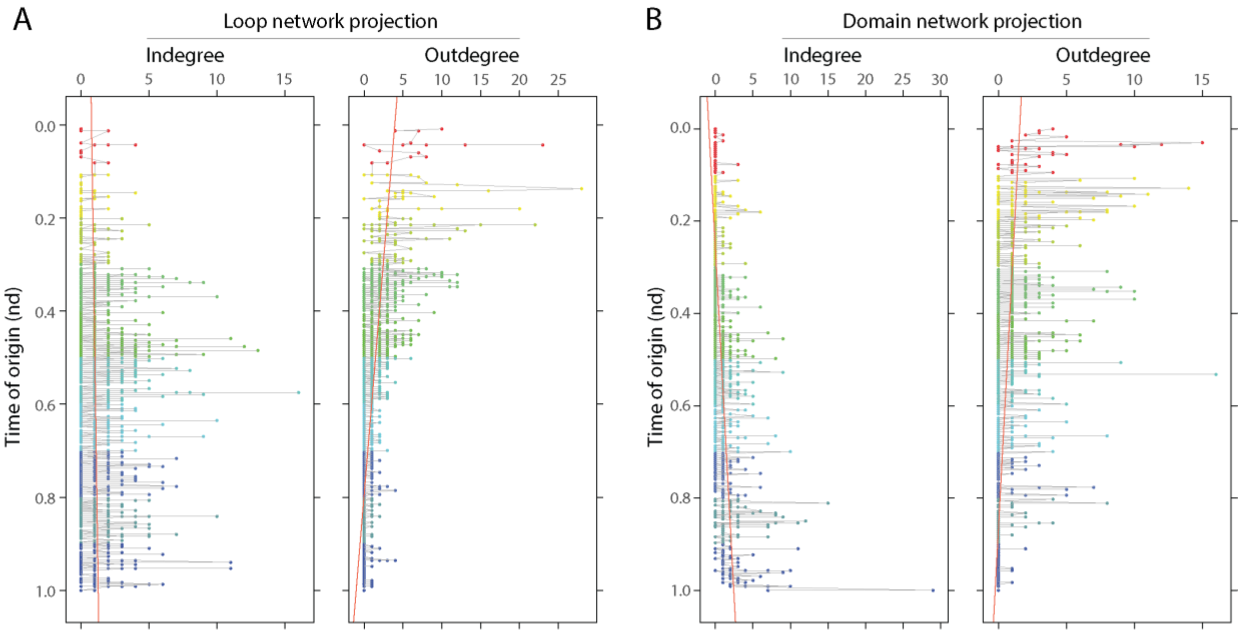

Figure S6. Connectivity of events along the timeline in the loop (**A**) and domain (**B**) network projections of the EF network. Data points describe indegree and outdegree of nodes of different ages of networks analyzed at present time (*nd* = 1). Symbols were color-coded according to node age. Linear regression lines (tomato-red) show rigorous trend of co-option of loops throughout the timeline and gradual growth of recruitment (embedding of loops) with age in emergent domains until recent times to cater for modern loop functionome (indegrees of panel **A** and **B**).

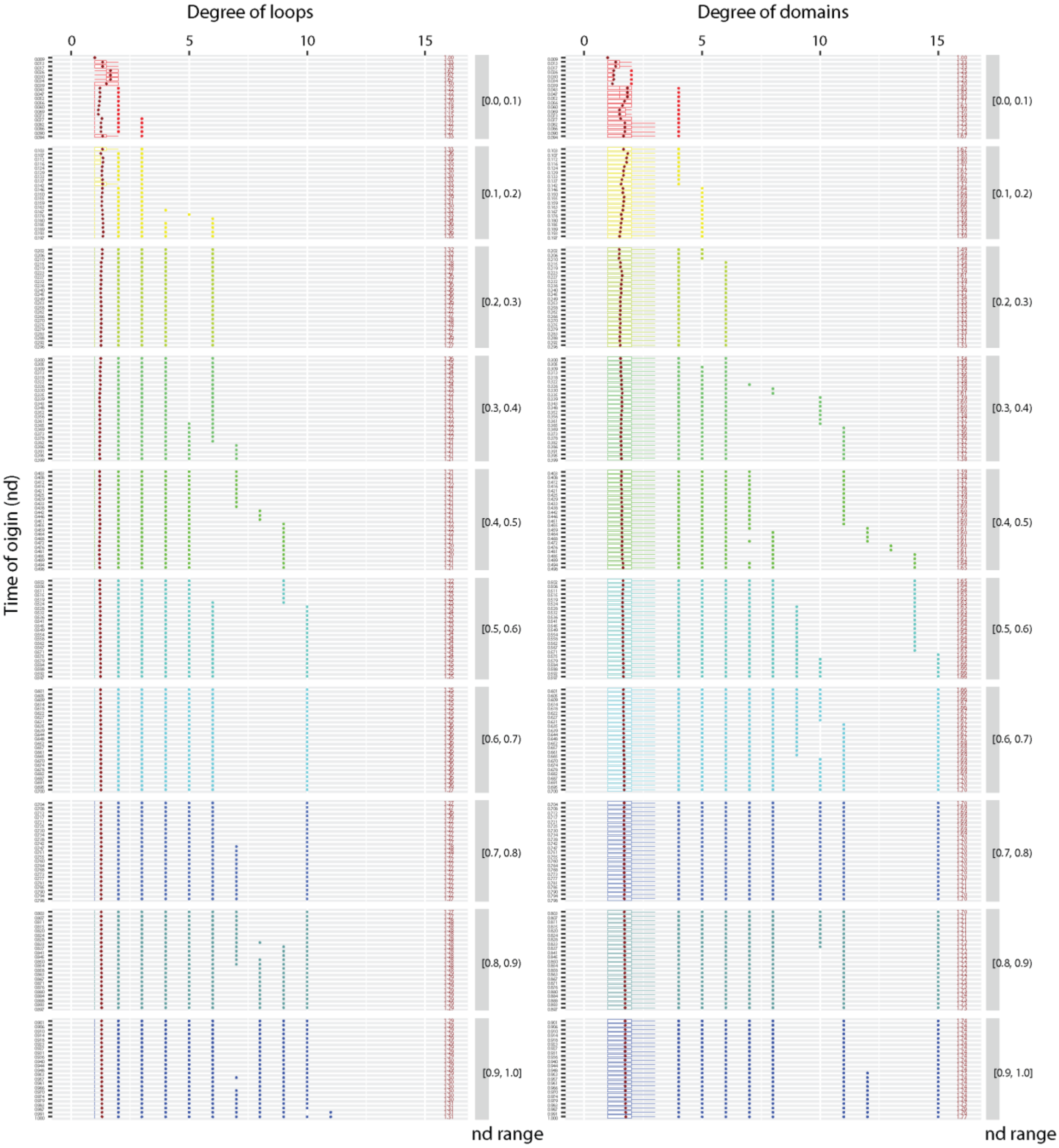

Figure S7. Measure of central tendency and dispersion in loop and domain components of the EF network. The accumulation (and dispersion) of node degrees of the bipartite network components with age (*nd* value) was captured through Tukey boxplots with boxes (first and third quartiles bracketing the median), whiskers (values within ±1.5 × inner quartile range), and outliers. Each boxplot describes the span of cumulative connectivity along events of the evolutionary timeline by providing visualization of chronological accumulation of undirected connections with time. Average values of connectivity of loops and domains were mapped using red diamonds and reported with two digits of significance. Each one of the aggregate 228 events along the timeline are labeled with their relative age in a 0-to-1 scale. The timeline of events was also coarse-grained into 10 age bins (colored red-to-blue).

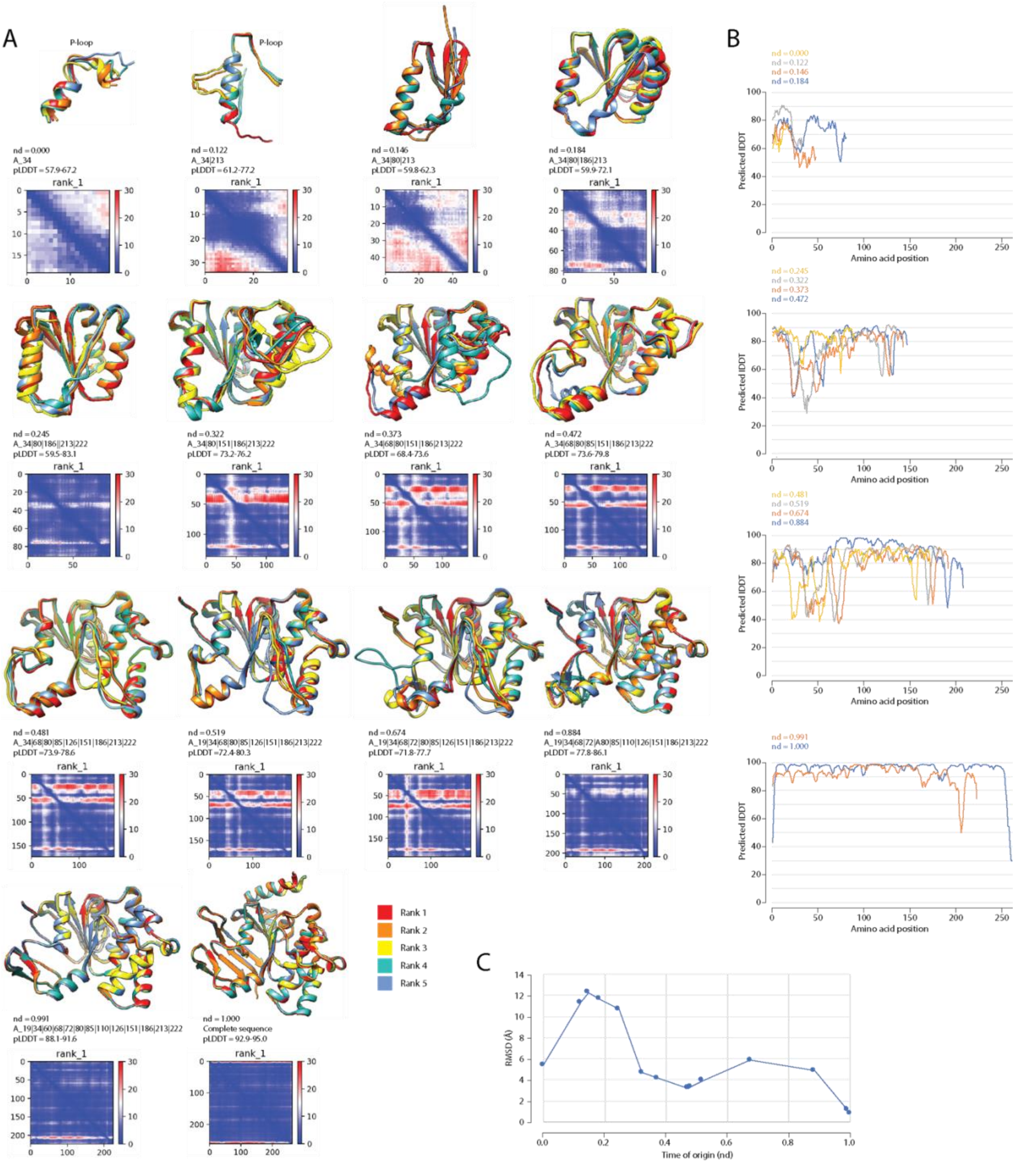

Figure S8. Confidence parameters of AlphaPhold2 structural predictions of a P-loop transporter. (**A**) Structural alignments of the 5 ranked predicted structures, pLDDT confidence levels, and predicted aligned error (PAE) plots that measure confidence in the relative position of residue pairs. (**B**) Predicted IDDT statistics at residue level for rank 1 predicted structures. (**C**) Pairwise structural alignments of predicted structures to the extant crystallographic entry (PDB 1B0U) show variation of RMSD values along the timeline.

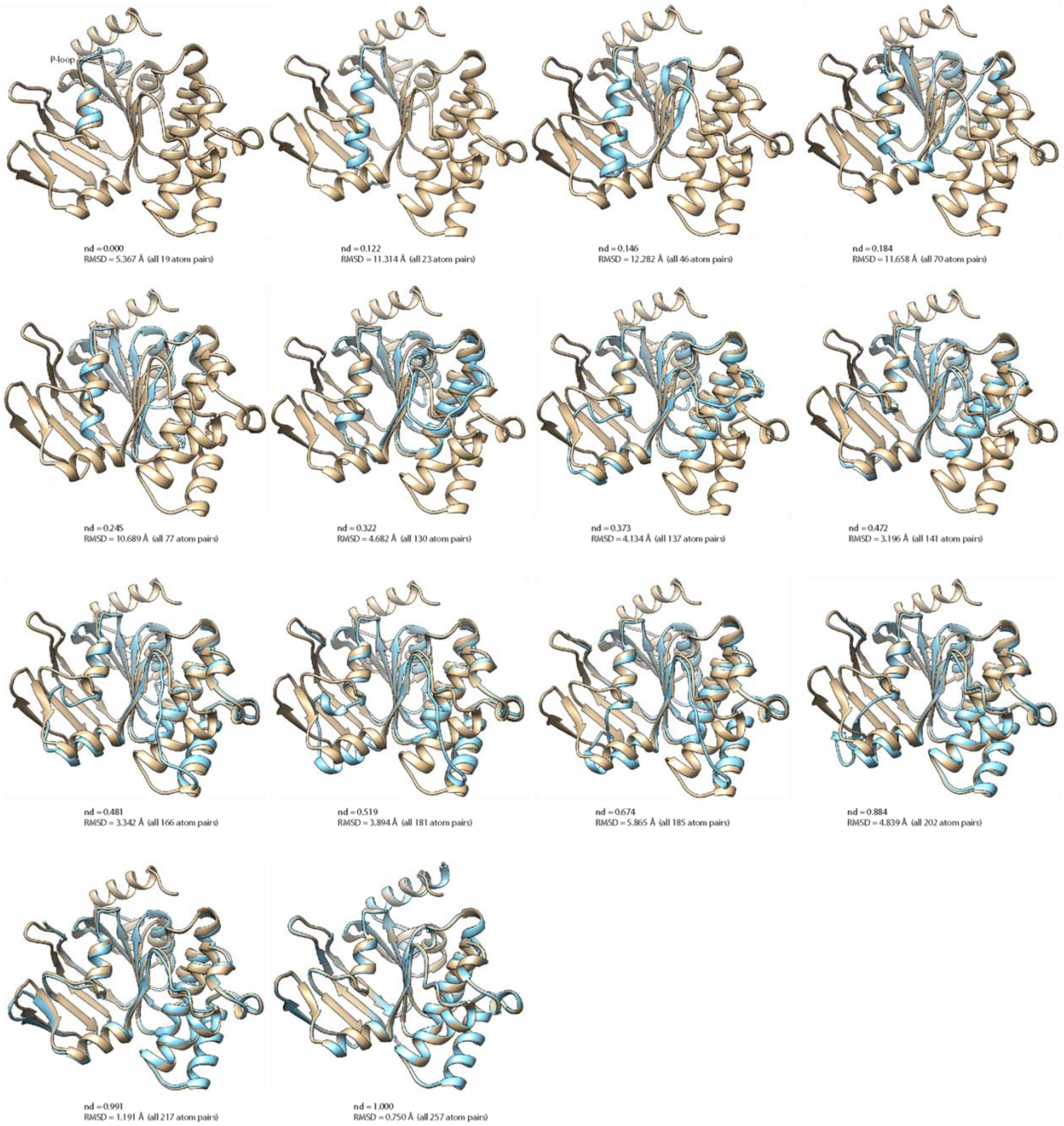

Figure S9. Structural alignment of modelled structures to the crystallographic entry of the P-loop transporter (PDB 1B0U).

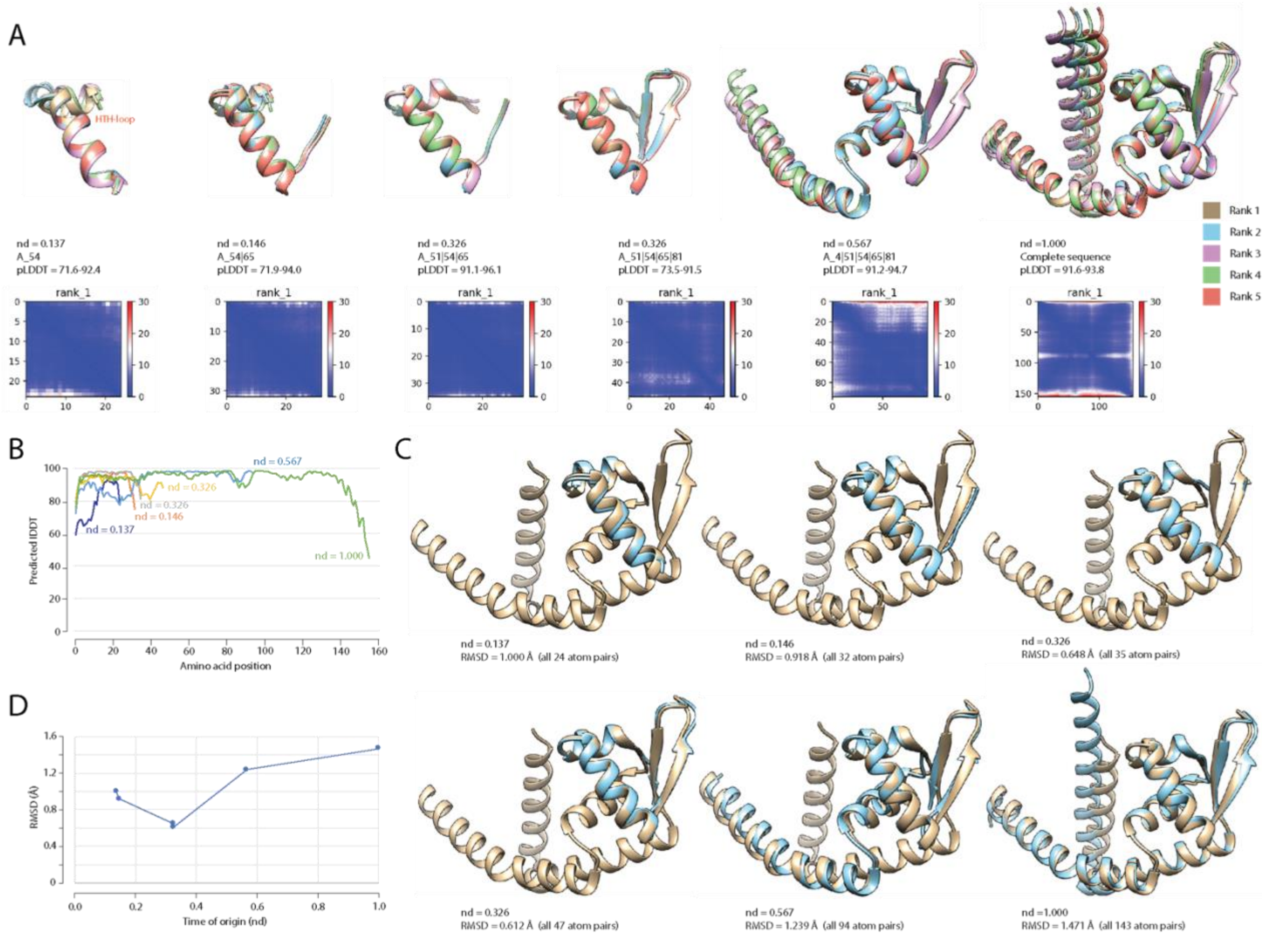

Figure S10. Confidence parameters of AlphaPhold2 structural predictions of the winged-helix transcriptional regulator mdtR. (**A**) Structural alignments of the 5 ranked predicted structures, pLDDT confidence levels, and predicted aligned error (PAE) plots that measure confidence in the relative position of residue pairs. (**B**) Predicted IDDT statistics at residue level for rank 1 predicted structures. (**C**) Pairwise structural alignment of modelled structures to the extant crystallographic entry of the winged-helix transporter (PDB 1S3J). (**D**) Variation of RMSD values for pairwise structural alignments along the timeline.

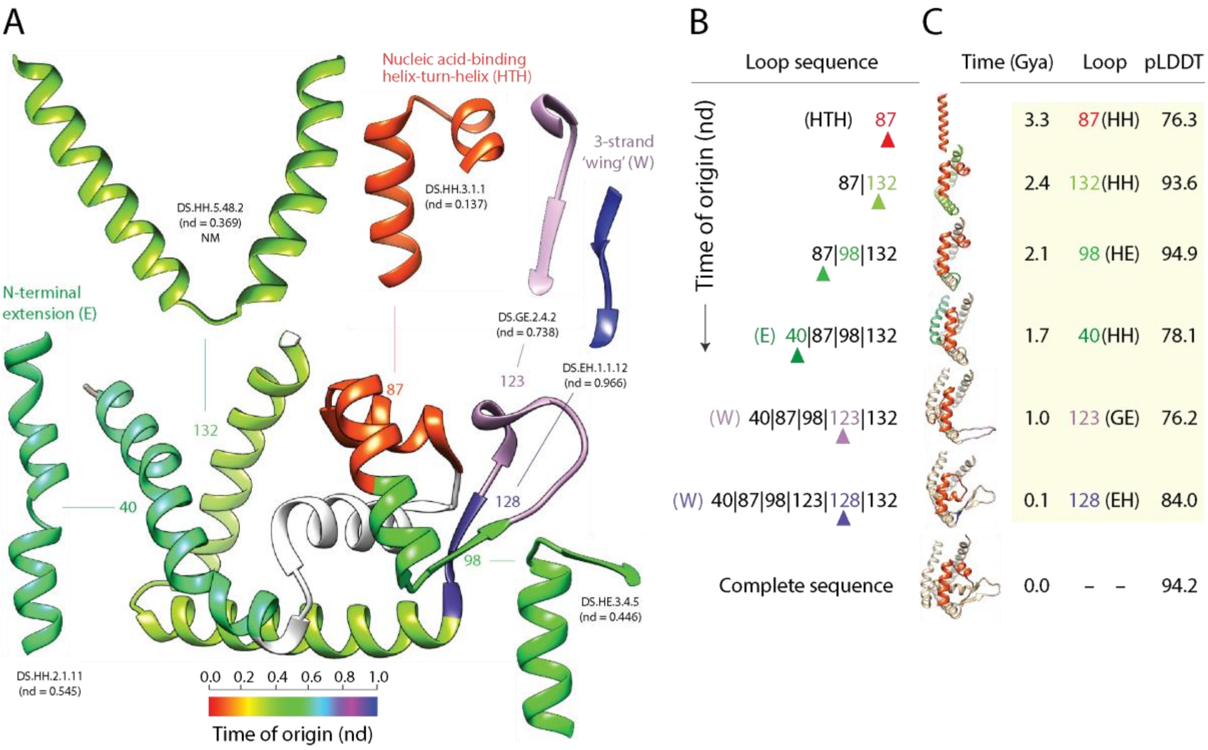

Figure S11. Tracing the evolutionary history of loop prototypes embedded in the structure of a variant of the primordial winged-helix domain. (**A**) A crystallographic model describing the atomic structure of the helix-turn-helix (HTH) containing nucleic acid-binding domain of a MarR complex of *Staphylococcus aureus* (PDB entry 4EM2) shows the nucleic acid-binding helix-turn-helix (HTH)-containing bundle packed against the 3-stranded β-sheet with 'wings' (W) and linked to an N-terminal extension. The different loop prototypes that make up the winged-helix domain structure are colored according to their time of origin, which is given as relative age (nd) in a scale from 0 (origin of proteins) to 1 (the present). (**B**) A time-ordered series of growing molecules was constructed by stitching loops together according to their time of origin. The sequence of loops is given from N- to C-terminus, with loops labeled in numbers and stitching interfaces indicated by pipe symbols. The last loop to be added to the sequence is indicated with an arrowhead and colored according to its age in each model. (**C**) Atomic structures of the growing molecules were modeled directly from their sequences with AlphaFold2. The age of the first loop (the HTH motif) and the last loop to be added to the structure are colored in the growing structures. The time of origin (Gya), loop number and makeup of bracing secondary structures (in parenthesis) of the newly added loop, and pLDDT confidence level of the *ab initio* prediction are given for each growing molecule.

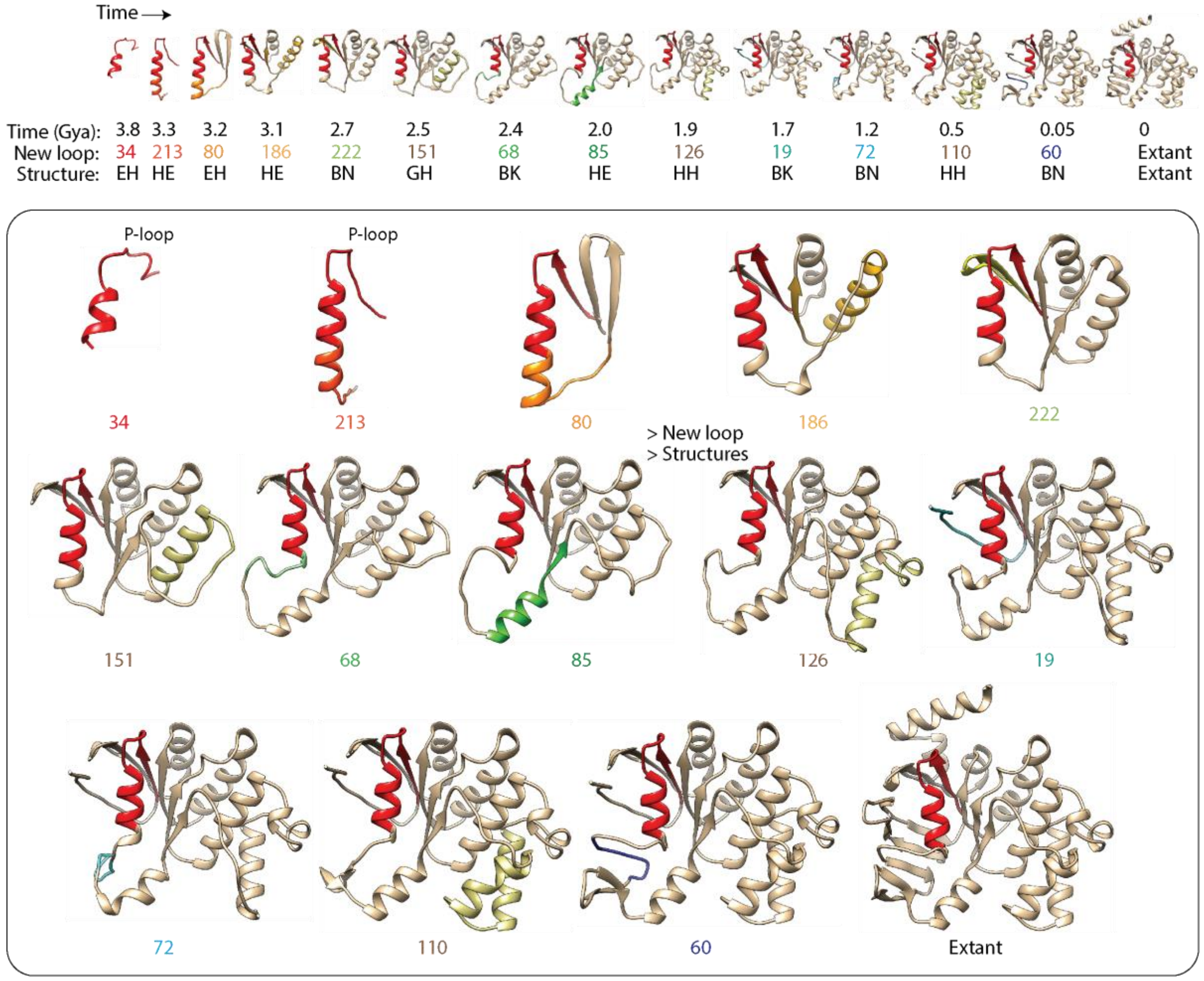

Figure S12. A time-ordered series of growing structures and corresponding atomic models of the P-loop containing ATP-binding domain. The figure complements Fig. 8 by showing the atomic structures of the growing molecules that were modeled directly from their sequences with AlphaFold2.

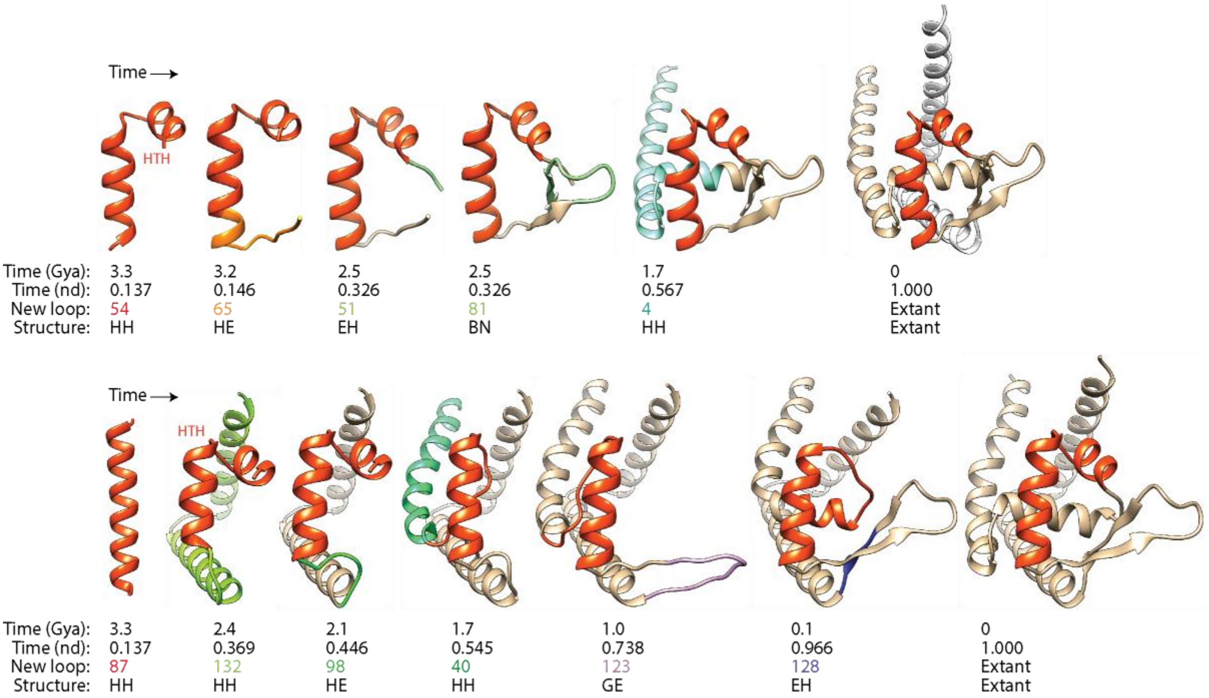

Figure S13. A time-ordered series of growing structures and corresponding atomic models of the winged-helix domain of two transcriptional regulators types. The figure complements Figs. 8 and S11 by showing the atomic structures of the growing molecules that were modeled directly from their sequences with AlphaFold2. The time series describes the growth of the winged-helix domain of the transcriptional regulators of the MarR (top) and mdtR (bottom) types.

Other Supplementary Material

Table S1. Loop-centric data.

Table S2. Domain-centric data.

Supplementary Video 1. Simulation of pairwise NG_{age} modularity of the EF network through progression of heat maps over the timeline, Nov 26, 2021.

Supplementary Video 2. Simulation of hierarchical organization of the EF network based on NG_{age} modularity through progression of dendrograms over the timeline, Nov 26, 2021.

Supplementary Video 3. Simulation of *waterfall* model of the loop network projection based on the linear (incremented by 1) progression of networks with nodes representing connectivity higher than or equal to various (0 to 100) percentiles of combined outdegree and indegree at Age 1, March 17, 2022.

Supplementary Video 4. Simulation of *waterfall* model of the domain network projection based on the linear (incremented by 1) progression of networks with nodes representing connectivity higher than or equal to various (0 to 100) percentiles of combined outdegree and indegree at Age 1, March 17, 2022.

Supplementary Video 5. Simulation of network growth with progression of node sizes and connectivity in the EF bipartite network over the evolutionary timeline, June 02, 2022.

Supplementary Video 6. Simulation of network growth with progression of node sizes and connectivity in the loop projection network over the evolutionary timeline, June 02, 2022.

Supplementary Video 7. Simulation of network growth with progression of node sizes and connectivity in the domain projection network over the evolutionary timeline, June 02, 2022.
